# Semantic design of functional de novo genes from a genomic language model

**DOI:** 10.1038/s41586-025-09749-7

**Published:** 2025-11-19

**Authors:** Aditi T. Merchant, Samuel H. King, Eric Nguyen, Brian L. Hie

**Affiliations:** 1 Department of Bioengineering, Stanford University, Stanford, CA USA; 2 Arc Institute, Palo Alto, CA USA; 3 Department of Chemical Engineering, Stanford University, Stanford, CA USA; 4 Stanford Data Science, Stanford University, Stanford, CA USA

**Keywords:** Machine learning, Protein design, Computational models, Genetic databases

## Abstract

Generative genomic models can design increasingly complex biological systems^1^. However, controlling these models to generate novel sequences with desired functions remains challenging. Here, we show that Evo, a genomic language model, can leverage genomic context to perform function-guided design that accesses novel regions of sequence space. By learning semantic relationships across prokaryotic genes^2^, Evo enables a genomic ‘autocomplete’ in which a DNA prompt encoding genomic context for a function of interest guides the generation of novel sequences enriched for related functions, which we refer to as ‘semantic design’. We validate this approach by experimentally testing the activity of generated anti-CRISPR proteins and type II and III toxin–antitoxin systems, including de novo genes with no significant sequence similarity to natural proteins. In-context design of proteins and non-coding RNAs with Evo achieves robust activity and high experimental success rates even in the absence of structural priors, known evolutionary conservation or task-specific fine-tuning. We then use Evo to complete millions of prompts to produce SynGenome, a database containing over 120 billion base pairs of artificial intelligence-generated genomic sequences that enables semantic design across many functions. More broadly, these results demonstrate that generative genomics with biological language models can extend beyond natural sequences.

## Abstract (web summary)

By learning a semantics of gene function based on genomic context, the genomic language model Evo autocompletes DNA prompts to generate novel genes encoding protein and RNA molecules with defined activities, whose sequences generalize beyond those found in nature.

## Main

**Fig. 1:**
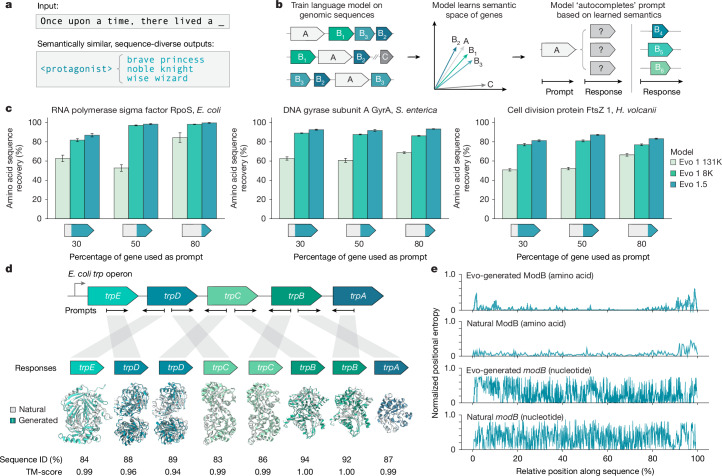
In-context genomic modelling and design with Evo. **a**, In natural language, distributional semantics holds that lexically distinct but functionally similar words often occur in similar contexts with shared sets of neighbouring words. **b**, In semantic design, a genomic language model trained across multiple genes learns to map genes with related functions to similar semantic spaces, enabling the generation of functionally related yet sequence-diverse genes. **c**, Sequence recovery assessments, where a genomic language model is used to autocomplete three conserved prokaryotic genes, show consistent improvements from Evo 1 131K and Evo 1 8K to Evo 1.5, reflecting an enhanced ability to leverage genomic context. The bar height denotes mean, and the error bars indicate standard error. *n* = 100 generated sequences. **d**, Completion of conserved *E. coli* *trp* operon gene sequences using both sense and antisense strand prompting yields high sequence recovery and predicted structural conservation in the generated sequences across the operon. TM, template modelling. **e**, A positional entropy comparison between natural and generated *modB* sequences at both the nucleotide and the amino acid level shows conservation of essential amino acid residues while maintaining high nucleotide diversity. *n* = 500 sequences.

Although generative artificial intelligence (AI) promises to accelerate the design of functional biological systems, articulating ‘function’ to a generative model remains challenging and often underspecified. In natural language, distributional semantics hypothesizes that meaning can be represented by word co-occurrence, that is, ‘you shall know a word by the company it keeps’^3,4^ (Fig. 1a). In biology, an emerging distributional hypothesis defines the function of a gene through its interactions with other genes, that is, ‘you shall know a gene by the company it keeps’^2^.

In prokaryotic genomes, functionally related genes are often positioned next to each other in gene clusters or operons^5,6^. Researchers have exploited this property, known as guilt by association, to characterize unknown genes neighbouring functionally characterized genes^7,8^, leading to discoveries of new molecular mechanisms and important biotechnological tools^9–12^. At its core, guilt by association leverages the distributional hypothesis of gene function for function-guided discovery.

A capable generative model of prokaryotic genomic sequences could learn this distributional notion of function to perform function-guided design. Progress in long-context machine learning has enabled generative models of genomic sequences at the multi-kilobase scale^1^. These models predict the next base pair in a sequence, enabling them to generate DNA sequences conditioned on a genomic sequence prompt (Fig. 1b).

Given the success of guilt by association, we reasoned that prompt engineering of a generative genomic model with a sequence of known function could direct the model to sample novel, functionally related sequences in its response. We call this approach ‘semantic design’, a generative strategy that harnesses the multi-gene relationships in prokaryotic genomes to design novel DNA sequences enriched for targeted biological functions. Of note, unlike traditional biological design, which involves combining or optimizing characterized sequences^13^, semantic design potentially allows for the exploration of new regions of functional sequence space.

Here we show that Evo, a genomic language model, learns a distributional semantics over genes that enables the function-guided design of new sequences reflecting prokaryotic functional relationships. We first demonstrate that Evo enables in-context genomic design, leveraging sequence conservation patterns to complete prokaryotic genes and operons.

We then apply semantic design to generate genes with high novelty and specified functional activity. First, we produce several novel toxin–antitoxin pairs with high experimental success rates, including a toxic gene with no significant sequence similarity to known bacterial toxins and a functional RNA antitoxin. We further generate multiple functional anti-CRISPRs (Acrs) that lack sequence or predicted structural similarity to known Acrs.

We also report SynGenome (https://evodesign.org/syngenome/), an AI-generated genomics database, containing over 120 billion base pairs of synthetic DNA sequences derived from prompts spanning 9,000 functional terms. We make SynGenome openly available to facilitate semantic design across diverse functions.

In summary, we demonstrate that semantic design, with its versatility and robust success rates, offers a promising framework for function-guided design that can generalize beyond observed evolutionary sequence landscapes.

## Evo enables in-context genomic design

To apply semantic design for function-guided sequence generation, a model must understand not just individual gene sequences, as with protein language models^14,15^, but also how genes relate to each other within broader genomic contexts. Evo, trained on prokaryotic DNA sequences from OpenGenome (Methods), processes long genomic sequences at single-nucleotide resolution^1^, enabling it to link nucleotide-level patterns to kilobase-scale genomic context. Given that functionally related sequences cluster on prokaryotic genomes, supplying appropriate genomic context as a prompt could condition Evo to generate novel genes whose functions mirror those found in similar natural contexts (Fig. 1a,b).

**Extended Data Fig. 1:**
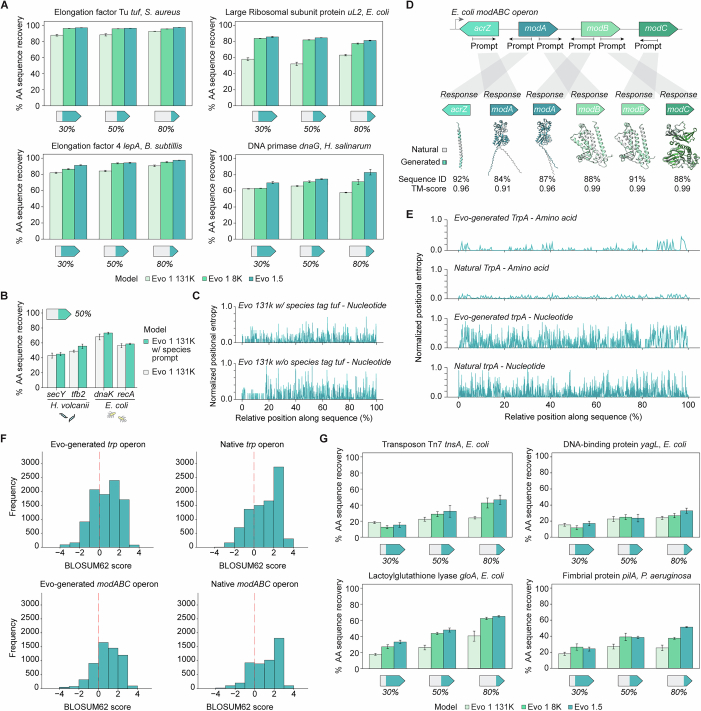
Additional analysis of in-context design using Evo. **A**, Bar graph showing sequence recovery of conserved genes *tuf*, *uL2*, *lepA*, and *dnaG* highlights the ability of Evo to understand genomic localization from prompts alone. AA, amino acid; bar height, mean; error bars, standard error; *n* = 100 generated sequences. **B**, Comparison of gene completion performance using Evo 1 131k with and without species tag prompt shows marginal improvements in gene completion ability with addition of species tag. AA, amino acid; bar height, mean; error bars, standard error; *n* = 50 generated sequences. **C**, Positional entropy analysis of Evo 1 131k-generated Elongation factor Tu (*tuf*) sequences with and without a species-tag prompt shows that the addition of species tag does not significantly alter generated sequence nucleotide entropy. *n* = 50 generated sequences. **D**, Completions of conserved *E. coli modABC* operon gene sequences in both forward and reverse directions demonstrate high sequence identity and structural conservation across the operon. TM-score, template modelling score. **E**, Positional entropy comparison between natural and generated *trpA* sequences at the nucleotide and amino acid levels shows conservation of essential amino acid residues while maintaining high nucleotide diversity. *n* = 100 sequences. **F**, BLOSUM62 score distributions comparing generated and native sequence variability for genes in the *trp* and *modABC* operons against *E. coli* K-12 reference genes show that Evo preferentially makes conservative amino acid substitutions. *n* = 100 sequences per gene. **G**, Gene completion performance across moderately (*gloA, pilA*) and poorly (*tnsA, yagL)* conserved *E. coli* genes exhibit poorer sequence recovery, in line with natural conservation patterns. AA, amino acid; bar height, mean; error bars, standard error; *n* = 50 generated sequences.

As an initial experiment, we assessed the ability of Evo to leverage genomic context by performing an ‘autocomplete’ task in which we prompted the model with partial sequences of highly conserved prokaryotic genes. We tested several archaeal and bacterial genes, including RNA polymerase sigma factor *rpoS* from *Escherichia coli*, DNA gyrase subunit A *gyrA* from *Salmonella enterica* and cell division protein *ftsZ1* from *Haloferax volcanii* (Fig. 1c and Extended Data Fig. 1a). We also tested three versions of the Evo model: Evo 1 8K (pretrained at 8,192 context length), Evo 1 131K (extended to 131,072 context length) and Evo 1.5, newly introduced here, which extends the pretraining of Evo 1 8K by 50% to 450 billion tokens (see Methods; Extended Data Fig. 1b,c). For each gene, we prompted the models with varying amounts (30%, 50% and 80%) of input sequence and evaluated their ability to complete the remainder.

Evo 1.5 consistently demonstrated the highest recovery of the natural protein sequence, particularly at lower prompt lengths. For instance, with just 30% of the input sequence, Evo 1.5 achieved 85% amino acid sequence recovery for *rpoS*, compared with 65% for Evo 1 131K. This performance advantage was maintained across all tested genes and prompt lengths, with Evo 1.5 achieving near-perfect sequence recovery at 80% input. These experiments align with previous findings that longer pretraining can improve learning of long-range interactions in sequence models^16–18^. We therefore selected Evo 1.5 for further investigation, and all results attributed to Evo in this study were produced by the Evo 1.5 model.

To further test Evo’s understanding of genomic context at a multi-gene scale, we next evaluated its ability to predict gene sequences based on operonic neighbours (Fig. 1d and Extended Data Fig. 1d). We prompted the model with sequences of genes either upstream or downstream of target genes in the *trp* and *modABC* operons, leveraging DNA complementarity to control directionality through sense or antisense strand prompts. Evo demonstrated robust predictive performance across all tested configurations, achieving over 80% protein sequence recovery for all target genes. Furthermore, the model exhibited adaptability to genomic orientation, generating upstream gene sequences when prompted with reverse complement of downstream genes, and vice versa. These results indicate that Evo not only learns the primary sequence of genes but also captures the broader genomic organization of bacterial operons.

To assess whether the generations from Evo went beyond trivial memorization of training sequences, we analysed the per-position entropy of both amino acid and nucleotide sequences in the model’s generations (Fig. 1e and Extended Data Fig. 1e). Using the *modABC* operon as a test case, we prompted the model with sequences encoding *modA* from *E. coli* K-12 and analysed variability in the generated *modB* responses. Amino acid-level entropy analysis revealed selective conservation, with generally lower entropy at key positions and higher variability in less-conserved regions, consistent with natural protein evolution. Further analysis of amino acid substitution patterns using BLOSUM62 showed that when the model generated sequences with amino acid changes, it preferentially selected conservative substitutions, mirroring natural evolutionary constraints (Extended Data Fig. 1f).

At the nucleotide level, we observed substantially higher entropy, with variation even in regions encoding conserved amino acids. We also observed lower sequence recovery in genes with low sequence conservation (Extended Data Fig. 1g). Given that a single prompt was used to generate all response sequences, these results suggest that Evo is not simply reproducing memorized sequences; rather, it is synthesizing information from across its training set, reflecting biological constraints while generating diversity in a manner reminiscent of natural evolution.

## Semantic design of multi-component systems

Next, we explored whether we could apply semantic design to biology with higher evolutionary variation: phage and bacterial defence systems. These systems, which are shaped by the evolutionary arms race between bacteria and phage^19^, are some of the most rapidly evolving systems in nature. Consequently, defence systems exhibit vast amounts of functional diversity and share limited sequence conservation across species^20,21^. Defence systems frequently cluster into defence islands, enabling the discovery of new systems through guilt-by-association approaches^22^.

**Fig. 2:**
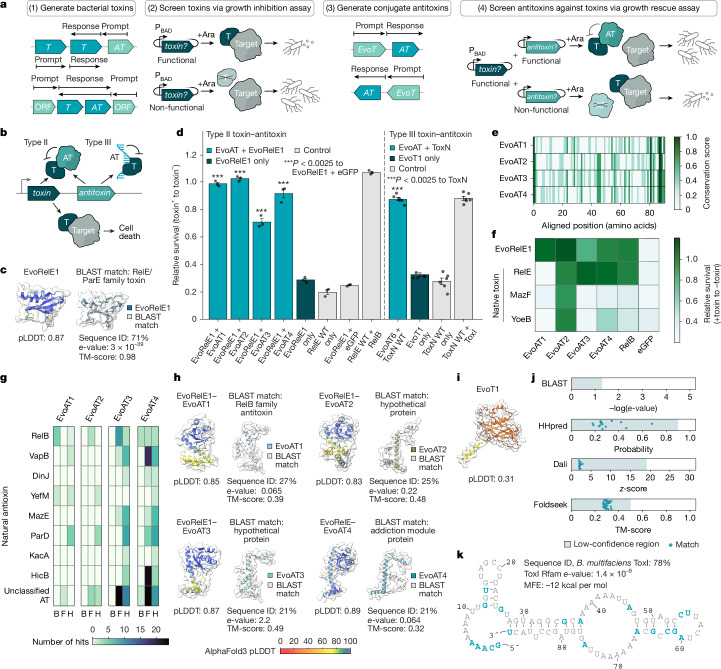
Evo generates functional toxin–antitoxin complexes with low similarity to nature. **a**, Semantic design of T2TA systems begins by generating toxins (Ts) from toxin genomic context prompts and testing growth inhibition. Functional toxins (EvoTs) then serve as prompts to generate cognate antitoxins (ATs), which are validated through growth recovery. Ara, arabinose; P_BAD_, *araBAD* promoter. **b**, T2TA (protein–protein) and T3TA (protein–RNA) systems function via antitoxin binding and neutralization of toxins. **c**, AlphaFold 3 structure comparison between the generated type II toxin EvoRelE1 and its closest BLAST match. **d**, Relative bacterial survival after 12 h when testing generated toxin–antitoxins, normalized to uninduced toxin controls. The type II antitoxins EvoAT1–4 rescue growth against EvoRelE1, whereas the type III antitoxin EvoAT6 rescues growth against ToxN. The bar height denotes the mean, the error bars indicate standard error, and the circles represent biological replicates (*n* = 3 for type II and *n* = 6 for type III). Significance was determined by a one-sided Student’s *t*-test against EvoRelE1 + eGFP (*P* = 1.6 × 10^−7^, 1.4 × 10^−7^, 2.3 × 10^−5^ and 1.7 × 10^−5^ for EvoAT1–4) and ToxN wild type (WT) only (*P* = 1.84 × 10^−8^) for type II and type III systems, respectively. **e**, Alignment of EvoAT1–4 amino acid sequences reveals discrete areas of conservation. **f**, Relative bacterial survival when testing EvoAT1–4 and RelB against natural type II toxins. EvoAT2 and EvoAT4 inhibit multiple natural toxins. **g**, Number of structural and sequence matches between EvoAT1–4 and natural antitoxin families. B, BLAST; F, Foldseek; H, HHpred. **h**, AlphaFold 3 structures of EvoAT1–4 in complex with EvoRelE1 and structural alignments of EvoAT1–4 to their closest BLAST matches. EvoAT1–4 have confident predicted structures despite limited sequence identity to natural proteins. **i**, AlphaFold 3 structure of the novel toxic protein EvoT1. **j**, Structural and sequence-similarity analyses of EvoT1 show no significant similarity to natural proteins. **k**, Minimum free energy (MFE) secondary structure and sequence of EvoAT6 show similarity to ToxI antitoxins. The blue lettering shows sequence differences.

Given the natural diversity and genomic colocalization of these systems, we sought to determine whether semantic design could be used to generate new defence systems. As an initial test case, we focused on type II toxin–antitoxin (T2TA) systems, some of which have a role in phage defence^23^. T2TAs consist of a toxin protein that inhibits bacterial growth or induces death under stress, paired with an antitoxin protein that binds and neutralizes the toxin in homeostatic conditions (Fig. 2a,b). These systems often maintain conserved genomic architectures despite sequence divergence, with toxin and antitoxin genes arranged in adjacent positions^24^.

**Extended Data Fig. 2:**
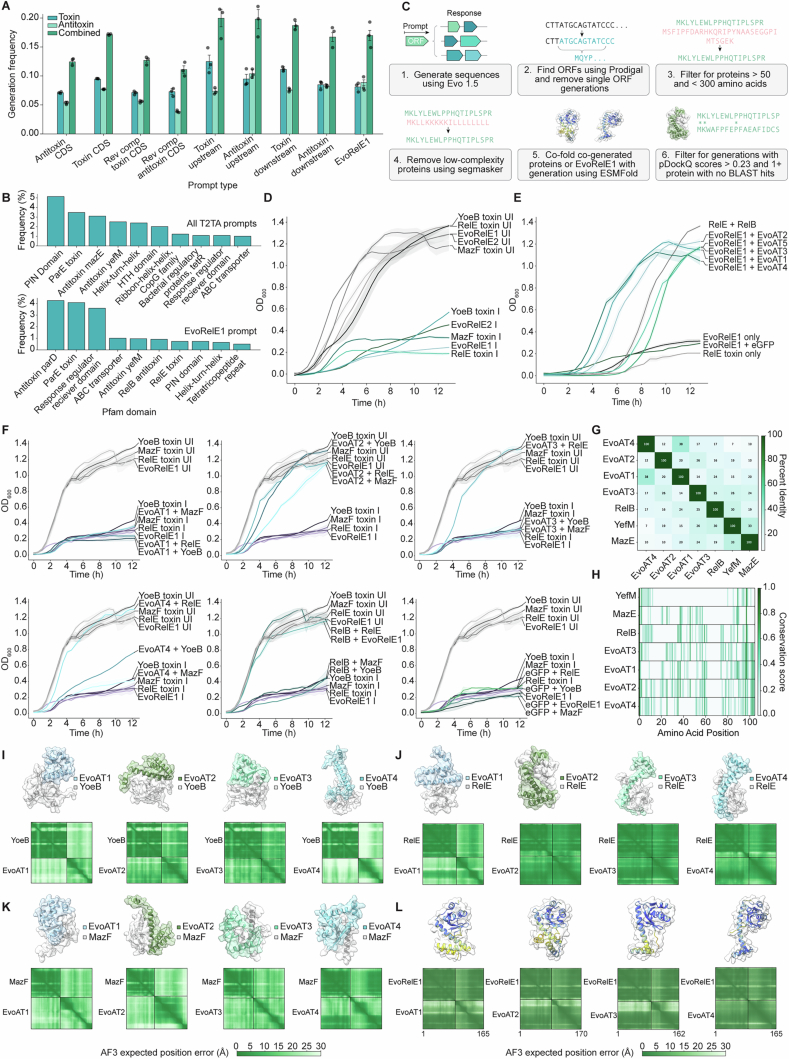
Semantic design of type II toxin-antitoxins systems. **A**, Frequency of toxin and antitoxin sequence generation for all eight prompt types post-pDockQ filtering. Prompting with the 500 bp upstream of natural toxin and antitoxin genes provides the highest frequency of T2TA sequence generation. Bar height, mean; error bars, standard deviation; circles, batches of generated sequences; *n* = 3. **B**, Most frequently generated Pfam domains for natural sequence-derived type II toxin-antitoxin prompts and EvoRelE1 prompt. Generations are enriched for type II toxin-antitoxin-related domains and families. **C**, Overview of the in silico filtering method used to select type II toxin and antitoxin candidates. **D**, Growth curves show growth arrest in *E. coli* when toxins EvoRelE1, EvoRelE2, and all natural toxins are induced. I, induced; UI, uninduced; line, mean; error shading, standard error; *n* = 3 biological replicates. **E**, Growth curves show growth rescue when EvoAT1-5 are present in combination with EvoRelE1 and growth arrest when only EvoRelE1 is present. I, induced; UI, uninduced; line, mean; error shading, standard error; *n* = 3 biological replicates. **F**, Growth curves of EvoAT1-4 with natural toxins YoeB, MazF, and RelE show growth rescue across all toxins for EvoAT2 and rescue against YoeB and RelE for EvoAT4. I, induced; UI, uninduced; line, mean; error shading, standard error; *n* = 3 replicate wells. **G**, Percent identity matrix for EvoAT1-4 against *E. coli* MazE, YefM, and RelB. EvoAT1-4 share limited overall sequence identity with wild-type antitoxins. **H**, Alignments of EvoAT1-4 against *E. coli* MazE, YefM, and RelB show some conserved residues in the C-terminus. **I-K**, AlphaFold 3 structures and predicted aligned error plots of Evo antitoxins in complex with YoeB (**I**), RelE (**J**), and MazF (**K**). (**L**) AlphaFold 3 predicted aligned error plots of EvoAT1-4 in complex with EvoRelE1 show generally confident interaction predictions.

To generate diversified T2TAs using Evo 1.5, we developed a prompting strategy that leveraged the colocalization of these systems (Fig. 2a). We curated eight types of prompts: toxin and antitoxin sequences, their reverse complements, and the upstream or downstream contexts of toxin and antitoxin loci (Extended Data Fig. 2a,b). Following sampling using these prompts, we filtered generations for sequences encoding protein pairs that exhibited in silico predicted complex formation (Methods). We also included a novelty filter, requiring at least one component to have only limited sequence identity to known T2TA proteins (see Methods; Extended Data Fig. 2c).

Using a growth inhibition assay (Methods), we were able to identify a functional bacterial toxin, EvoRelE1, that exhibited strong growth inhibition (approximately 70% reduction in relative survival) while possessing 71% sequence identity to a known RelE toxin (Fig. 2c,d and Extended Data Fig. 2d).

We subsequently prompted Evo 1.5 with the sequence of EvoRelE1, hypothesizing that the model could use the context of the toxin to generate conjugate antitoxins (Fig. 2a). Following sampling, we found that generated sequences were enriched for antitoxin-like genes (Extended Data Fig. 2b), demonstrating that context could guide generation towards desired functional outcomes. After filtering generations using the same criteria as for toxins (Methods), we identified ten antitoxin candidates with minimal sequence identity to natural proteins.

Following co-expression with EvoRelE1, 50% of the Evo-generated antitoxin candidates rescued cell growth (Fig. 2d and Extended Data Fig. 2e). The most effective candidates, EvoAT1 and EvoAT2, restored growth to 95–100% of normal cell survival, with candidates EvoAT3 and EvoAT4 demonstrating moderate rescue activity (70% and 90% relative survival, respectively). Sequence alignments of the successful antitoxins revealed discrete regions of conservation (Fig. 2e), potentially highlighting motifs required for toxin neutralization despite their overall sequence diversity. Furthermore, when tested against natural RelE, MazF and YoeB toxins, several of the generated antitoxins were able to rescue growth across multiple toxins, with EvoAT2 showing inhibitory activity against all three toxins and EvoAT4 rescuing growth against RelE and YoeB (Fig. 2f and Extended Data Fig. 2f). In contrast, the natural RelB antitoxin only neutralized its cognate RelE toxin and EvoRelE1. This finding is notable given that the EvoATs share limited overall sequence identity with the antitoxin counterpart of each toxin (Fig. 2g and Extended Data Fig. 2g,h). When co-folded using in silico structure prediction methods, several of the EvoATs had low-confidence predicted complex formation with the natural toxins that they inhibited (Extended Data Fig. 2i–k), highlighting the potential for semantic design to generate molecular interactions not readily identifiable by structure prediction models.

EvoAT1 through EvoAT4 all had relatively minimal sequence identity to natural proteins (21–27%), a range in which sequence similarity alone can make it difficult to predict shared function^25,26^. The closest direct sequence matches for EvoAT2 and EvoAT3 were to proteins not annotated as antitoxins, with EvoAT2 showing 25% sequence identity to an uncharacterized *Magnetococcus* sp. YQC-5 hypothetical protein (*e*-value of 0.06) and EvoAT3 showing 21% sequence identity to a *Jatrophihabitans* hypothetical protein (*e*-value of 2.2; Fig. 2h). As these natural proteins appear in T2TA-like genomic contexts (Supplementary Fig. 1), our results suggest that they may function as part of toxin–antitoxin systems. These findings illustrate how Evo’s understanding of genomic context may help with functional annotation of previously uncharacterized proteins, as well as functional design that is unconstrained by existing annotations.

**Extended Data Fig. 3:**
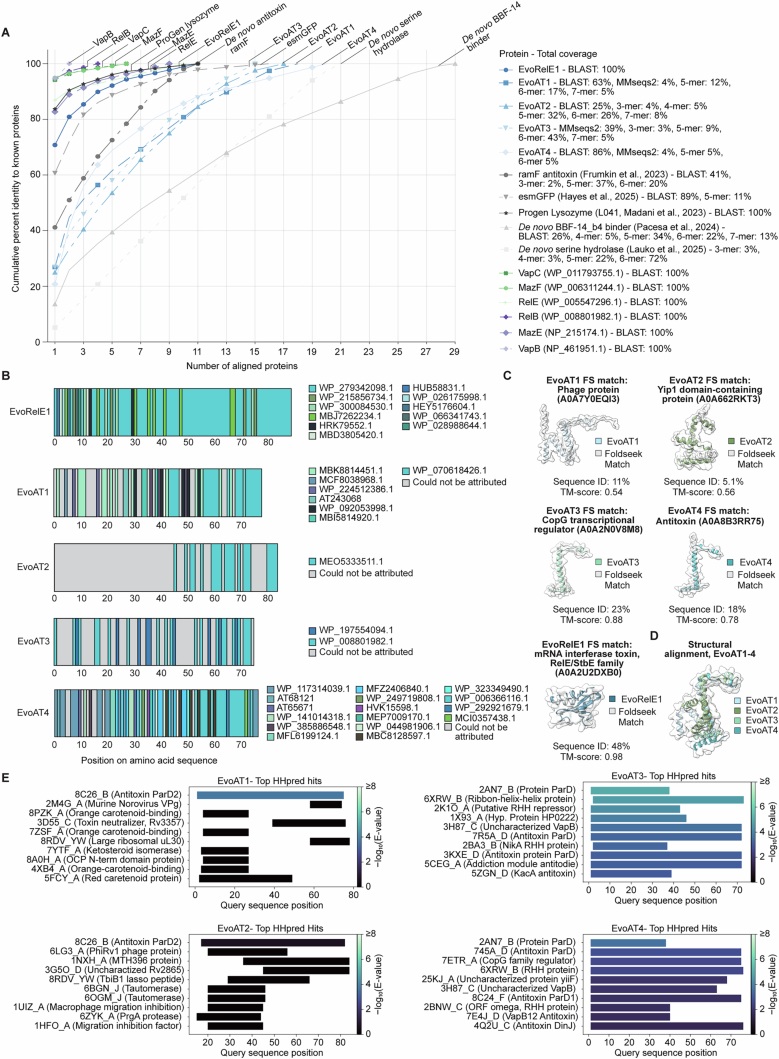
Bioinformatic characterization of semantically designed type II toxins and antitoxins. **A**, Cumulative protein residue attribution analysis of EvoAT1-4 and EvoRelE1 compared to natural, language model-generated, and de novo designed proteins. EvoAT1-4 are substantially diversified, requiring more proteins to reach full-coverage than other language model designed proteins. **B**, BLAST and MMseqs2-based per-residue attribution of EvoAT1-4 and EvoRelE1. EvoRelE1 and EvoAT4 show high degrees of compositional chimaerism, with residue attributions to over ten proteins each. EvoAT1-3 have substantial unattributed regions, highlighting the diversity of their sequences. **C**, Structural alignments of the AlphaFold 3 predicted structures of EvoAT1-4 and EvoRelE1 with their closest Foldseek matches show generally high alignment with natural proteins. TM-score, template-modelling score. **D**, Structural alignment of the AlphaFold 3 structures of EvoAT1-4 shows somewhat similar structures, with a generally aligned C-terminus. **E**, Top HHpred hits for EvoAT1-4. All generated EvoATs show some similarity to natural antitoxins despite high degrees of sequence divergence.

Structural predictions for EvoAT1 and EvoAT2 co-folded with EvoRelE1 had high confidence (predicted local distance difference test (pLDDT) scores of 0.85 and 0.83, respectively; Fig. 2h) and exhibited minimal predicted position error (Extended Data Fig. 2l). This, coupled with the strong functional activity of EvoAT1–4, was particularly noteworthy given the limited sequence identity of these antitoxins to known antitoxins (Fig. 2h). To further assess the novelty of EvoAT1–4, we performed a residue coverage analysis to determine the number of natural proteins required to account for each amino acid position (Methods). Although natural toxins and antitoxins could be constructed by recombining 2–6 different natural proteins, EvoAT1–4 required fragments from 15–20 different proteins (Extended Data Fig. 3a,b). Overall, these results further underscore the ability of Evo to generate functional proteins with low similarity to natural proteins.

More sensitive sequence and structural similarity searches using BLAST, HHpred, Dali and Foldseek (Methods) revealed that the EvoATs showed similarity to multiple independent antitoxin superfamilies, particularly ParD, MazE, HicB and VapB (Fig. 2g and Extended Data Fig. 3c–e). This finding, coupled with the activity of EvoAT2 and EvoAT4 against multiple toxins, is notable because their cognate toxins use different mechanisms of action^27–30^. This suggests that Evo may have identified a broader functional compatibility between antitoxins and toxins than is typically observed in nature, highlighting the potential of semantic design to provide new insights into protein–protein interaction compatibility^31^.

To evaluate utility of semantic design for systems containing non-coding RNAs, we next focused on type III toxin–antitoxin systems (T3TAs). Like type II systems, T3TAs maintain a conserved genomic architecture of adjacent toxin and antitoxin genes. However, instead of an antitoxin protein, T3TA systems include a repetitive RNA antitoxin that directly binds to the toxin protein, repressing toxins in homeostasis^32^ (Fig. 2b).

**Extended Data Fig. 4:**
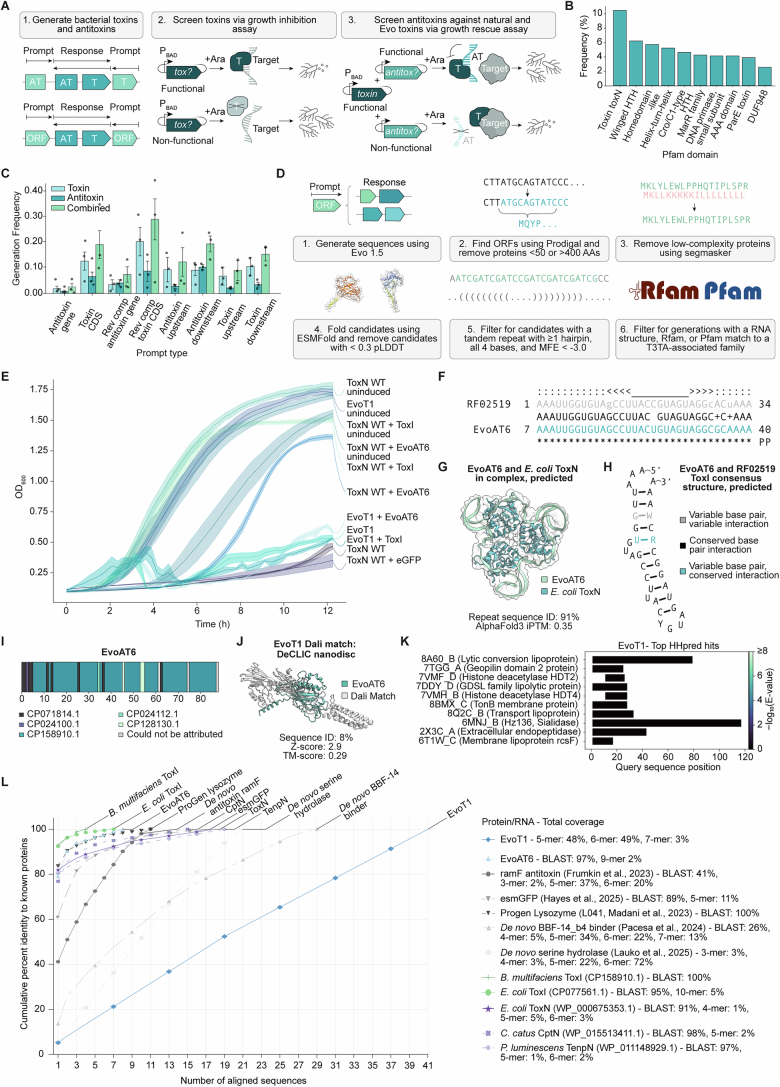
Semantic design of type III toxin-antitoxin systems. **A**, Semantic design of type III toxin-antitoxin systems begins by generating toxins (T) and antitoxins (AT) from type III system genomic context prompts. Toxins are tested for growth inhibition, after which functional toxins and natural toxins are tested against generated antitoxins for growth recovery. Ara, arabinose; P_BAD_, *araBAD* promoter. **B**, Most frequently generated Pfam domains for type III toxin-antitoxin prompts. Generations are enriched for type III toxin-related domains. **C**, Frequency of toxin and antitoxin sequence generation for all prompt types post-ESMFold filtering. Prompting with toxin-related sequences results in higher success rates. Bar height, mean; error bars, standard deviation; circles, batches of generated sequences; *n* = 3. **D**, Overview of the in silico filtering method used to select type III toxin-antitoxin candidates. **E**, Growth curves show bacterial growth arrest when EvoT1 is induced and growth rescue when EvoAT6 is co-transformed with toxN. Line, mean; error shading, standard error; *n* = 6 biological replicates. **F**, Alignment of the consensus repeat sequence of EvoAT6 with the ToxI Rfam consensus repeat shows generally aligned dot-bracket structures despite sequence differences. **G**, AlphaFold 3 trimer structure of EvoAT6 in complex with *E. coli* ToxN. iPTM score, interface predicted template modelling score. **H**, Consensus secondary structures of EvoAT6 and ToxI repeat motifs show generally similar secondary structures. **I**, BLAST and MMseqs2-based per-residue attribution of EvoAT6. EvoAT6 appears to be primarily composed of five distributed ToxI sequences. **J**, Alignment between the AlphaFold 3 structure of EvoT1 and its closest Dali match shows generally weak alignment. TM-score, template modelling score. **K**, Top HHpred hits for EvoT1. EvoT1 has generally weak alignments to proteins unrelated to toxin-antitoxin systems. **L**, Cumulative sequence attribution analysis of EvoT1 and EvoAT6. EvoT1 requires similar numbers of proteins to reach full-coverage as de novo proteins, while EvoAT6 requires more sequences to reach full-coverage than natural ToxI genes.

Using a similar prompting approach to that of the T2TAs, we used Evo 1.5 to sample sequences with prompts derived from individual T3TA genes, their reverse complements and their respective upstream and downstream sequences (Extended Data Fig. 4a–c). To identify candidate sequences, we filtered generations for sequences containing a complex tandem repeat sequence and at least one RNA structure, Rfam or Pfam match to a T3TA-associated family (see Methods; Extended Data Fig. 4d).

Using a growth rescue assay of generated RNA antitoxin candidates against wild-type (WT) type III toxins ToxN, TenpN and CptN (Extended Data Fig. 4a), we identified an Evo-generated antitoxin candidate, EvoAT6, that had neutralizing activity (88% relative survival) against ToxN^33^ (see Methods; Fig. 2d and Extended Data Fig. 4e). This antitoxin showed only moderate sequence identity to known type III antitoxins, with 78% identity to ToxI from *Bacillus* *multifaciens* (Fig. 2k and Extended Data Fig. 4f). Despite this sequence divergence, the predicted consensus repeat secondary structure of EvoAT6 closely resembled that of the natural ToxI sequence, indicating that our design approach successfully diversified the antitoxin while preserving essential structural features (Extended Data Fig. 4g–i).

Using T3TA prompts, we also semantically designed a toxic protein, EvoT1, that showed strong growth inhibition (33% relative survival) upon expression in *E. coli* (Fig. 2d,i). EvoT1 was not neutralized by EvoAT6 or natural antitoxins (Extended Data Fig. 4e), suggesting diverse mechanisms among our generated sequences. Of note, EvoT1 showed no strong sequence or predicted structural similarity to known bacterial toxin–antitoxin genes, even when using sensitive similarity detection methods (Fig. 2j and Extended Data Fig. 4j,k). A residue coverage analysis (Methods) required recombining segments from over 40 proteins to account for all amino acids in EvoT1, which more closely resembled the compositionality of de novo proteins than either natural proteins or sequences designed by protein language models (Extended Data Fig. 4l). In summary, these results demonstrate that semantic design can generate multi-component systems containing proteins and RNA with high degrees of novelty and functional specificity.

## Semantic design of de novo anti-CRISPRs

**Fig. 3:**
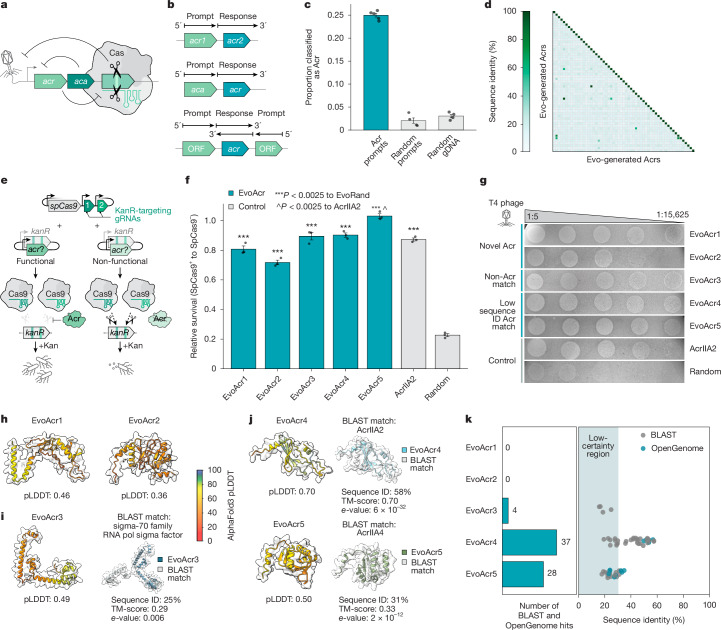
Evo generates functional anti-CRISPR proteins with no clear similarity to known proteins. **a**, Type II anti-CRISPR systems feature anti-CRISPR (*acr*) genes encoding inhibitors of type II-A Cas nucleases that often co-occur with anti-CRISPR-associated genes (*aca*). **b**, Semantic design of Acrs uses known type II Acr genomic contexts as prompts. **c**, PaCRISPR classification shows significant enrichment of Acr-like sequences in generations from Acr prompts. The bar height denotes the mean, the error bars represent standard error, and the circles show batches of generations (*n* = 4 generation batches). gDNA, genomic DNA. **d**, Sequence identity matrix demonstrates high diversity among randomly sampled generated Acrs. **e**, In protection assays, functional Acrs block SpCas9 cleavage of a kanamycin resistance gene, enabling cell survival in kanamycin-supplemented media. kanR, kanamycin resistance gene. **f**, Relative bacterial survival after 8 h when testing generated Acrs normalized to uninduced SpCas9 controls. EvoAcr1–5 confer protection against SpCas9, with EvoAcr3–5 showing comparable activity with AcrIIA2. The bar height denotes the mean, the error bars show the standard error, and the circles represent biological replicates (*n* = 3). Significance was determined by a one-sided Student’s *t*-test compared with random control (*P* = 8.3 × 10^−6^, 6.2 × 10^−6^, 8.2 × 10^−6^, 1.4 × 10^−6^ and 8.2 × 10^−7^ for EvoAcr1–5, respectively) and AcrIIA2 (*P* = 5.1 × 10^−4^ for EvoAcr5). **g**, T4 phage plaque assays validating Acr activity. Plaque formation indicates successful Acr protection. The experiment was performed in triplicate with representative images shown. **h**, AlphaFold 3 structures for EvoAcr1–2 show low-confidence predictions. **i**, AlphaFold 3 structures comparing EvoAcr3 and its closest BLAST match show limited sequence and structural similarity to a sigma-70 family protein. **j**, AlphaFold 3 structures comparing EvoAcr4–5 with their closest BLAST matches show moderate structural and sequence similarity to known Acr proteins. **k**, Sequence similarity analysis of EvoAcr1–5 against BLAST nr and OpenGenome. EvoAcr1–2 demonstrate no significant sequence identity to known proteins, EvoAcr3 exhibits sequence similarity to proteins at percent identities too low for reliable functional inference, and EvoAcr4–5 share limited sequence identity with known Acrs.

We next explored whether semantic design could generate sequences with even greater evolutionary novelty. Anti-CRISPRs (Acrs) are proteins used by phages to neutralize bacterial CRISPR–Cas systems (Fig. 3a). Many Acrs represent striking examples of rapid protein evolution, appearing as novel innovations without detectable similarity to other protein families^34–36^. Their diversity spans varied mechanisms of action, ranging from direct Cas binding and DNA mimicry to transcriptional silencing^37–40^, making them valuable tools for understanding protein evolution and developing control systems for CRISPR^41^.

Despite this diversity, many Acr operons maintain a somewhat conserved architecture, consisting of multiple *acr* genes appearing together alongside anti-CRISPR-associated (*aca*) genes^42^ (Fig. 3b). This architectural conservation makes Acrs an ideal test case for assessing the ability of the semantic design to generalize with respect to sequence while retaining a desired higher-level function.

**Extended Data Fig. 5:**
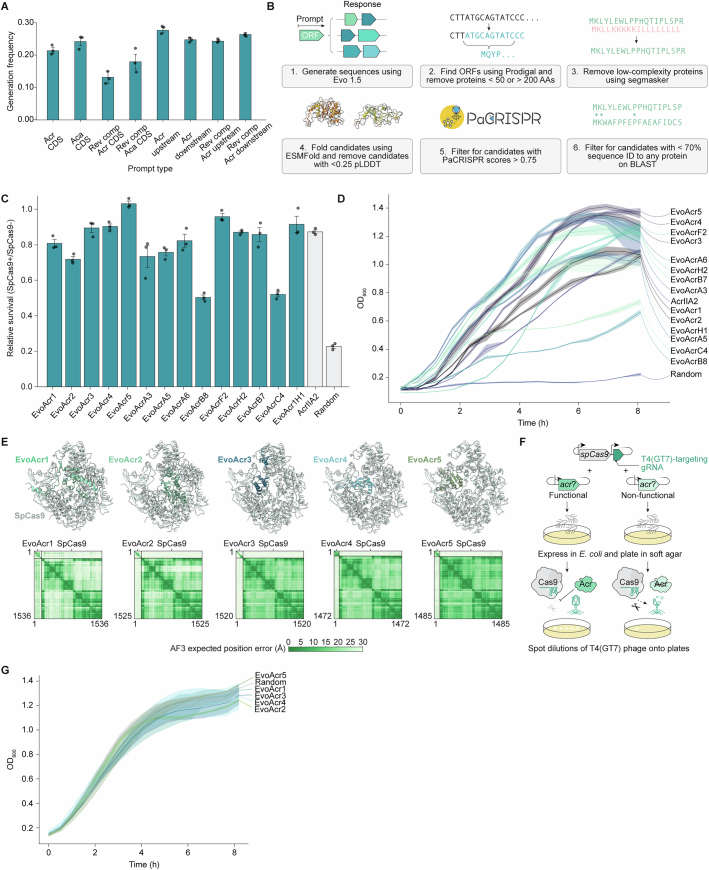
Experimental data related to semantic design of anti-CRISPRs. **A**, Frequency of anti-CRISPR generation for all eight prompt types post-ESMFold filtering as assessed by PaCRISPR predictions. Prompting with the upstream and downstream context of known Acrs results in a higher frequency of anti-CRISPR generation. Bar height, mean; error bars, standard deviation; circles, batches of generated sequences; *n* = 3. **B**, Overview of the in silico filtering method used to select anti-CRISPR candidates. **C**, Bar plot showing relative survival rates for all successful generated Acrs. Bar height, mean; error bars, standard error; circles, biological replicate values; *n* = 3 replicates. **D**, Growth curves indicate growth rescue in the presence of functional EvoAcrs and growth arrest in the presence of non-functional sequences. Line, mean; error shading, standard error; *n* = 3 biological replicates. **E**, AlphaFold 3 structure predictions of Evo Acrs in complex with SpCas9 (top) and their corresponding predicted aligned error (pAE) plots (bottom). **F**, To evaluate the activity of EvoAcrs in an orthogonal manner, a phage plaque assay was used in which functional Acr proteins prevent SpCas9-mediated cleavage of T4(GT7) phage, enabling successful phage infection and plaque formation at lower dilutions of phage. **G**, Growth curves of bacteria transformed with only EvoAcr1-5 show similar uninhibited growth patterns in line with a random sequence control. Line, mean; error shading, standard error; *n* = 3 replicate wells.

To semantically design Acrs, we leveraged sequences from known Cas9-targeting Acr operons as prompts (see Methods; Fig. 3b and Extended Data Fig. 5a), including type II *acr* genes, their associated *aca* genes, the 500 bp upstream and downstream of each *acr* gene and the reverse complements of both gene types. After filtering for size, complexity and structure (Extended Data Fig. 5b), we next used PaCRISPR, a machine learning model trained to identify potential Acr proteins, to evaluate our generated candidates^43^. Consistent with our prompts providing successful functional conditioning, generations derived from Acr-containing genomic contexts were substantially more likely to be classified as potential Acrs by PaCRISPR than negative control sequences (Fig. 3c and Extended Data Fig. 5a). Furthermore, the distribution of sequence identities among the predicted Acrs showed a wide range of novelty, with most candidates showing low similarity to each other (median pairwise sequence identity of 23%; Fig. 3d). This enrichment for diverse Acr-like sequences suggests that semantic design can bias generations towards a desired function even in the absence of clear sequence conservation.

To test the protection ability of Acr candidates against SpCas9, we co-transformed *E. coli* with plasmids encoding candidate Acrs and a CRISPR-targeted kanamycin resistance gene, where functional Acrs would preserve kanamycin resistance by inhibiting CRISPR-mediated cleavage^44^ (Fig. 3e). We found that 17% of tested sequences exhibited measurable Acr activity (Extended Data Fig. 5c,d), a notably high success rate given the lack of structural priors or conditioning (Extended Data Fig. 5e) and the use of a single Cas nuclease for screening. From this pool, we further identified five proteins (EvoAcr1–5) that demonstrated strong protection against SpCas9 cleavage in both liquid culture survival assays (Fig. 3f and Extended Data Fig. 5d) and phage infection experiments (Fig. 3g and Extended Data Fig. 5f), while maintaining normal host growth (Extended Data Fig. 5g).

**Extended Data Fig. 6:**
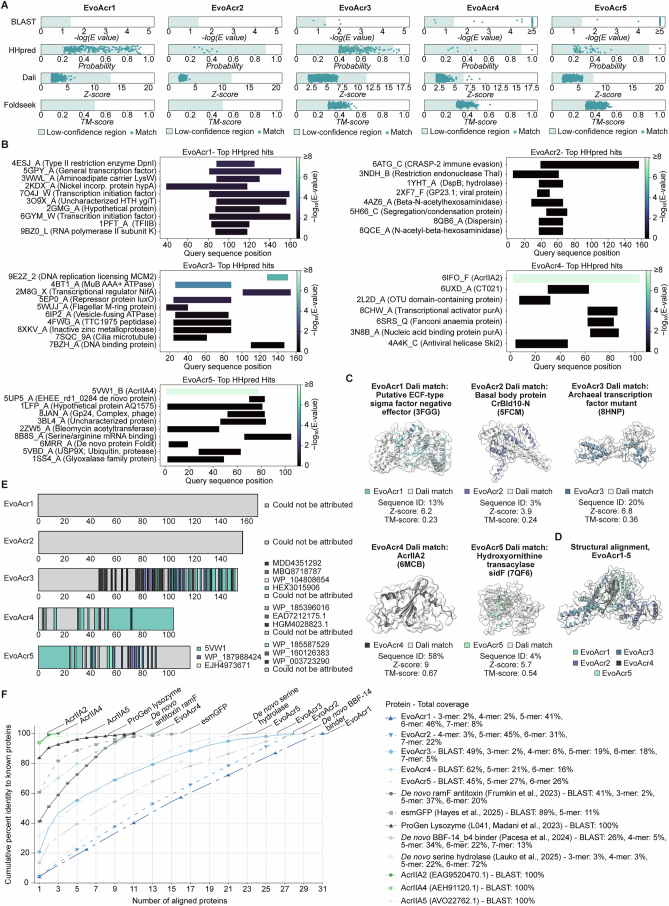
Bioinformatic characterization of semantically designed anti-CRISPRs. **A**, Distribution of matches following sequence and structural characterization of EvoAcr1-5 using HHpred, Dali, BLAST, and Foldseek shows generally few significant hits to known proteins. **B**, Top HHpred hits for each EvoAcr protein. EvoAcr1 and EvoAcr2 have weak moderate-to-low coverage hits to non-anti-CRISPR proteins while EvoAcr3 has moderate coverage hits to transcription factor and transposition-related proteins. **C**, Structural alignments of the AlphaFold 3 structures of EvoAcr1-5 and their closest Dali matches. TM-score, template modeling score. **D**, Structural alignment of the AlphaFold 3 structures of EvoAcr1–5 reveals minimal structural similarity. **E**, BLAST and MMseqs2-based per-residue attribution of EvoAcrs. EvoAcr1 and EvoAcr2 have no significant attributable segments while EvoAcr3-5 display moderate attribution to known proteins. **F**, Cumulative protein residue attribution analysis of EvoAcr1-5 compared to natural, language model-generated, and de novo designed proteins. EvoAcr1 and EvoAcr2 require similar numbers of proteins to reach full coverage as de novo proteins.

Detailed bioinformatic analysis of these five Acrs revealed a high level of sequence diversity. EvoAcr4 and EvoAcr5 shared moderate sequence similarity to known Acrs, with 58% and 31% sequence identity to AcrIIA2 and AcrIIA4, respectively (Fig. 3j and Extended Data Fig. 6a). Both demonstrated robust protection against SpCas9, showing activity comparable with the positive control AcrIIA2 in liquid culture assays (relative survival rates of 0.91 and 1.01 out of 1.0, respectively, compared with 0.87 for AcrIIA2; Fig. 3f). EvoAcr3 presented an intriguing case: although sharing limited sequence and predicted structural alignment (sequence ID = 25%, *E* = 0.006, template modelling score = 0.29) with a sigma-70 family RNA polymerase sigma factor, it maintained strong Acr activity (relative survival of 0.89; Fig. 3f,i). Further characterization using HHpred (Methods) revealed moderate-coverage alignments to various DNA-binding proteins, none of which were previously associated with Acr activity (Extended Data Fig. 6a,b). This suggests a potential mode of CRISPR inhibition that is not well characterized in existing functional databases.

Most notably, EvoAcr1 and EvoAcr2 represented proteins that eluded both sequence and structural characterization, showing no significant sequence identity to proteins in OpenGenome or BLAST nr (Fig. 3h,k). Further characterization of EvoAcr1 and EvoAcr2 using Dali (Methods) found no strong structural alignments to natural proteins (Extended Data Fig. 6a,c), although the low confidence scores of the predicted structures limited the reliability of this structural comparison (Fig. 3h and Extended Data Fig. 6d). In addition, a residue-level compositionality analysis (Methods) revealed that EvoAcr1 and EvoAcr2 required pieces from 28 and 31 different natural proteins, respectively, to achieve full sequence coverage (Extended Data Fig. 6e,f). This level of novelty is comparable with established de novo proteins such as RFdiffusion-generated serine hydrolases (21 proteins)^45^ and BindCraft-generated BBF-14 binders (29 proteins)^46^ and is substantially more novel than natural Acrs (2–6 proteins; Extended Data Fig. 6f).

Using more sensitive methods such as HHpred to characterize EvoAcr1 and EvoAcr2 also produced limited significant results, with EvoAcr1 having only low-coverage matches to proteins not thought to be Acrs and EvoAcr2 having no significant matches (Extended Data Fig. 6a,b). Despite this lack of similarity to known Acrs, both EvoAcr1 and EvoAcr2 demonstrated robust protection in both liquid culture and phage infection assays, with relative survival rates of 0.82 and 0.74 (out of 1.0), respectively. This experimental validation of novel, functional Acrs supports the ability of semantic design to leverage learned genomic organization patterns to access unexplored regions of sequence space. Together with EvoAcr3–5, these results demonstrate that semantic design can guide the generation of diverse Acr proteins, from variants of natural proteins to entirely new sequences.

## SynGenome: 120 gigabases of Evo-generated DNA

Following our validation that semantic design can generate functional proteins from genomic context alone, we reasoned that semantic design could be applied to create genes from across prokaryotic biology. To this end, we developed SynGenome, a database of synthetic DNA sequences designed using Evo. Applying the principles underlying semantic design, we prompted the model with 1.7 million prokaryotic and phage genes, generating sequences encompassing the broad functional diversity encoded in prokaryotic genomes.

**Fig. 4:**
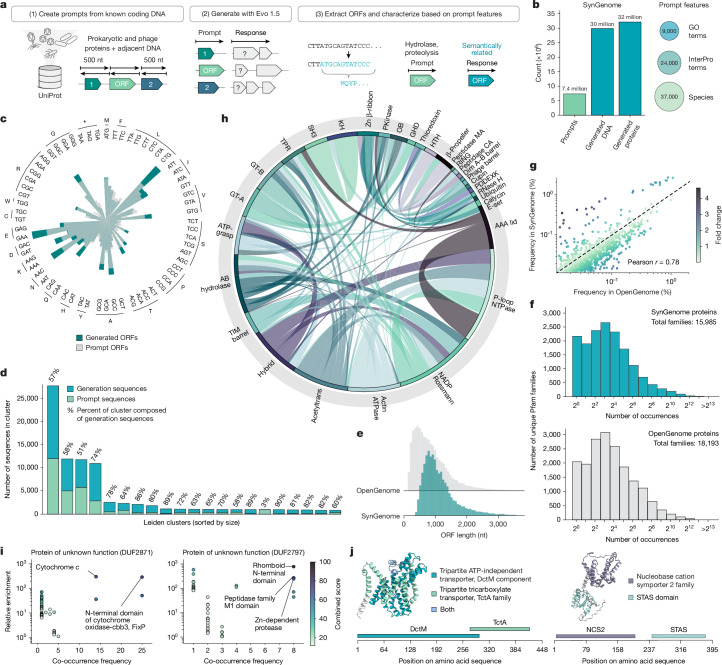
120 billion base pairs of AI-generated genomic sequences with SynGenome. **a**, To construct SynGenome, we derived prompts from known protein-coding genes, generated synthetic DNA sequences using Evo and bioinformatically characterized the generated sequences. **b**, Number of prompts and generated sequences in SynGenome, along with their associated features. GO, Gene Ontology. **c**, Codon usage patterns in a representative sample of Prodigal-predicted ORFs from prompt and generated sequences show preservation of codon preferences (*n* = 36,762 sequences per sample). **d**, Bar plot showing relative proportions of generation and prompt sequences in the most populous Leiden clusters, with percentages indicating the fraction of generated sequences per cluster. **e**, Distribution of Prodigal-predicted ORF lengths for representative samples of OpenGenome and SynGenome sequences. ORFs in SynGenome follow natural-looking length distributions. *n* = 36,762 sequences per sample. nt, nucleotide. **f**, Distribution of the number of Pfam protein family occurrences in representative samples of SynGenome and OpenGenome. Both follow a similar long-tailed distribution of family abundance. *n* = 36,762 sequences per sample. **g**, Scatterplot showing individual Pfam protein family frequencies in SynGenome and OpenGenome. Frequencies appear to be correlated, suggesting preservation of natural protein family abundance patterns. **h**, Circos plot showing the most enriched connections between Pfam clans found in natural sequence-derived prompts and generated responses across SynGenome. **i**, Scatterplots showing the most enriched prompt–response associations for two example DUFs in SynGenome. DUF2871 associates with cytochrome *c* and cytochrome oxidase domains, whereas DUF2797 associates with the rhomboid N-terminal domain, peptidase family M1 domain and Zn-dependent protease domains, consistent with previously hypothesized roles and domain associations for these DUFs^48,49^. **j**, Examples of chimeric proteins found in SynGenome representing potentially novel fusions of protein domains (Extended Data Fig. 7g,h).

To construct SynGenome, we leveraged the UniProt database to identify protein-coding genes and their adjacent sequences from prokaryotic organisms and bacteriophages. For each coding sequence, we extracted six distinct prompts: the upstream region, coding sequence, downstream region and their respective reverse complements (Fig. 4a). Using the Evo 1.5 model, we generated multiple synthetic sequences for each prompt, resulting in a database containing over 120 billion DNA base pairs (Methods).

To facilitate functional exploration of the database, we organized the generated sequences according to the Gene Ontology and InterPro domain annotations of their corresponding prompts^47,48^ (Fig. 4b), expecting enrichment for functionally related elements. After removing low-complexity sequences (Methods), we also used ESMFold to obtain predicted structures of 3.7 million putative protein-coding genes, enabling structure-based downstream analyses.

**Extended Data Fig. 7:**
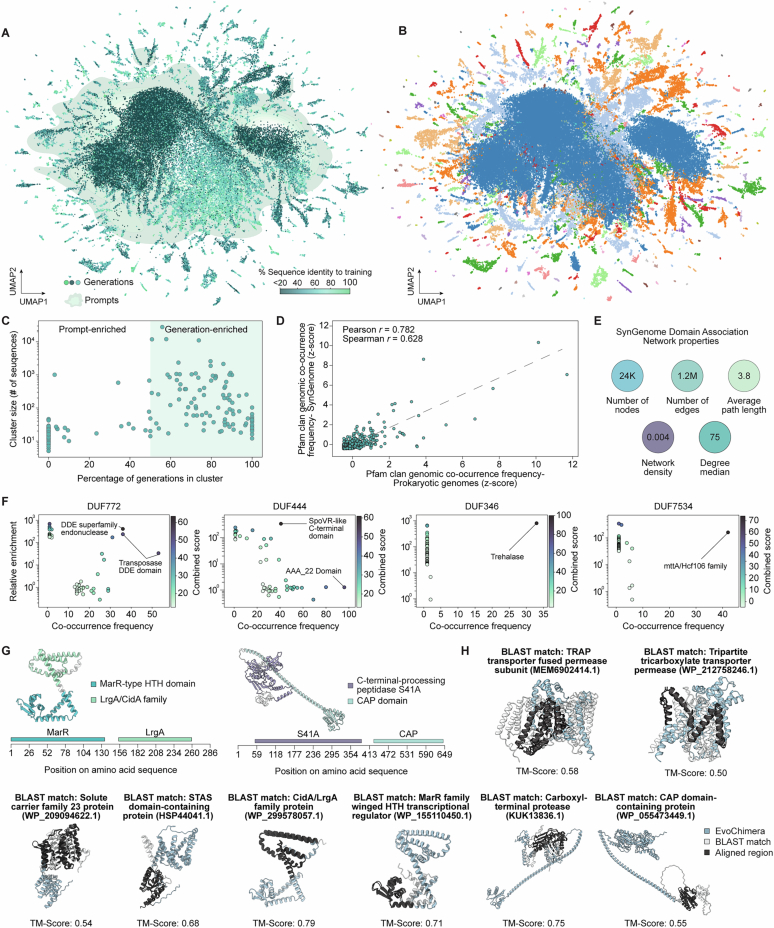
Extended analysis of SynGenome proteins and captured functional associations. **A**, UMAP of jointly projected Evo embeddings of SynGenome and prompt sequences. Generation embeddings are colored by sequence identity to training data and prompt embeddings are shown in light green. **B**, UMAP coloured by the Leiden clusters of the Evo embeddings of prompt sequences and generated sequences. **C**, The distribution of prompts and generations across Leiden clusters shows generally mixed clusters with both prompt and generated sequences present, with some smaller clusters containing only prompts or only generated sequences. **D**, Scatterplot showing correlation of prompt-response Pfam clan associations in SynGenome and syntenic Pfam clan associations in a representative group of prokaryotic genomes. Captured associations in SynGenome correlate well with natural syntenic relationships. **E**, Properties of the SynGenome domain association network. **F**, Additional scatterplots showing the most enriched prompt-response associations for domains of unknown function (DUFs) in SynGenome. DUF772 associates with transposition-related DDE domains, DUF444 associates with SpoVR, DUF346 associates with a trehalase, and DUF7534 associates with a sec-independent translation-related domain. These associations are consistent with previously hypothesized roles and known domain architectures related to these DUFs^48,103,104^. **G**, Additional examples of chimeric proteins found in SynGenome. **H**, Structural alignments of chimeric proteins found in SynGenome against their closest natural protein sequence matches. TM-score, template modelling score.

To characterize SynGenome, we first examined codon usage patterns between generated sequences and prompts. This analysis revealed that generated sequences closely mirrored prompt sequences, maintaining similar codon preferences (Fig. 4c). We further examined the prompt–generation relationships in Evo embedding space by performing Leiden clustering (Fig. 4d and Extended Data Fig. 7a,b). As expected, most clusters contained a mix of prompt and generated sequences, indicating that generations and natural sequences generally occupied similar regions of embedding space. However, we observed that 54 clusters (19% of the generated sequences) consisted primarily of generated sequences, potentially highlighting synthetic sequences that extended beyond the semantic space occupied by natural sequence embeddings (Extended Data Fig. 7c).

When compared with sequences from OpenGenome, we found that SynGenome-generated open reading frames (ORFs) followed the natural prokaryotic ORF length distribution (Fig. 4e). At the protein level, SynGenome matched natural Pfam domain frequencies globally (Fig. 4f) and individually (Pearson correlation coefficient *r* = 0.78; Fig. 4g). These analyses demonstrate that SynGenome recapitulates the general characteristics and protein diversity of natural prokaryotic sequences.

To probe the functional relationships captured by SynGenome, we constructed and analysed a network linking protein families in SynGenome prompts to those in generated responses (Methods). We found that the protein clans in prompt–response pairs mirrored natural genomic colocalization patterns (Fig. 4h and Extended Data Fig. 7d,e), further supporting that genomic conditioning can guide the generation of functionally related responses. We then investigated whether the functional associations captured by SynGenome could provide functional hypotheses for domains of unknown function (DUFs). As one example, we observed that DUF2871 strongly co-occurred with ‘cytochrome *c*’ (co-occurring in 14 prompt–response pairs) and ‘N-terminal domain of cytochrome oxidase-cbb3, FixP’ (co-occurring in 25 prompt–response pairs), consistent with previous structural hypotheses linking DUF2871 to cytochrome *c* proteins^49^ (Fig. 4i and Extended Data Fig. 7f). These findings demonstrate that the genomic associations captured in SynGenome not only recapitulate known colocalization relationships but may also aid with function prediction. We have provided an interactive visualization of this network (https://evodesign.org/syngenome/network), which may also serve as a valuable tool to identify appropriate prompts for functions of interest.

In addition to capturing known domain associations, SynGenome also contains several chimeric proteins (Fig. 4j and Extended Data Fig. 7g,h) with potentially novel domain fusions not widely known to exist in natural proteins. These fusions could represent new functional combinations or provide insights into unexplored protein architectures, opening avenues for designing proteins with new or enhanced functional properties.

**Extended Data Fig. 8:**
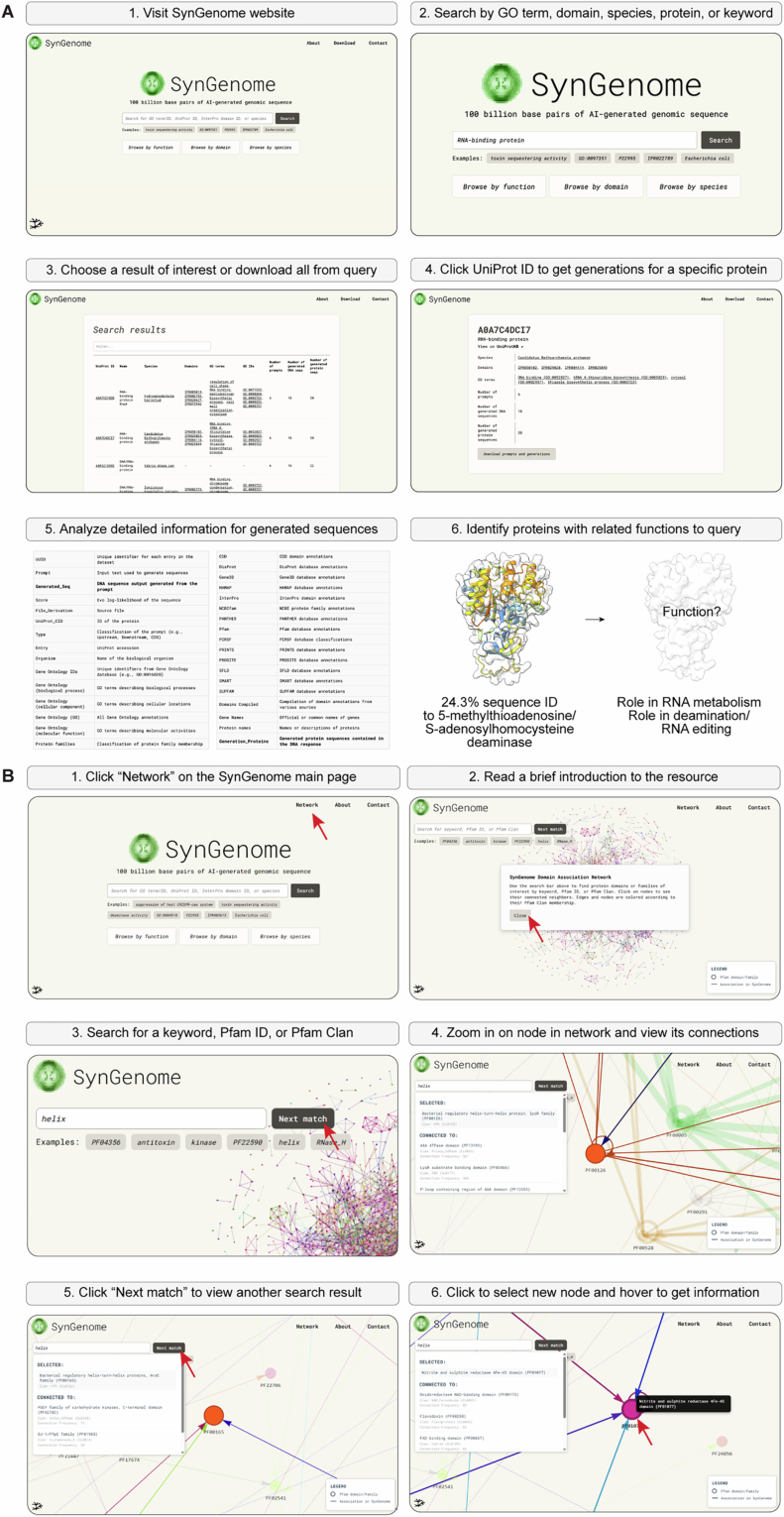
Example walkthrough of the SynGenome web-interface workflow. **A**, SynGenome can easily be queried with a domain, function, species, or keyword of interest to find relevant generated sequences, which can then be downloaded to identify potential functionally related protein candidates. We demonstrate the applicability of SynGenome by showing its generation of a potential RNA modification enzyme in response to an RNA-binding protein-derived prompt. **B**, The SynGenome domain association network can be searched for a Pfam family name, ID, or keyword of interest to see all connected domains to a relevant node found in prompt-response pairs across SynGenome. Displayed information also includes the number of times the association was seen across SynGenome and the Pfam clan membership of the selected and connected nodes.

Together, these results highlight the potential for SynGenome to become a valuable tool for exploring and expanding protein function through semantic relationships. By searching through SynGenome, researchers could discover functionally related proteins that extend beyond natural sequence space, gain insights into potential functions of uncharacterized genes, and create diverse screening libraries for exploring context-informed functions (Extended Data Fig. 8a,b).

SynGenome, including all 120 billion generated base pairs and 3.7 million predicted structures, is freely available (https://evodesign.org/syngenome/). The database is searchable with protein names, UniProt IDs, InterPro domains, species names or Gene Ontology terms of interest. We anticipate that SynGenome can serve as a practical tool that facilitates gene discovery and engineering with semantic design for the broader scientific community.

## Discussion

Advanced genomic sequence models, trained on hundreds of billions of DNA base pairs across prokaryotic life, can enable powerful capabilities for understanding and engineering biological systems. Here, we demonstrate that Evo enables controllable design of desired functions encoded in prokaryotic genomes by leveraging natural genomic contexts, achieving high experimental success rates of 17–50% when testing just tens of variants and surpassing success rates of many protein design methods^15,45^. Many of the designed proteins have no significant sequence identity to proteins of a similar function or, in some cases, to any known protein. These results blur the line between de novo protein design^50–52^ and diversification based on evolutionary models^14,15,53^, providing an ‘existence proof’ that sequence models can meaningfully generalize beyond natural sequences.

Semantic design represents a fundamentally new approach for protein design that is complementary to existing approaches. First, unlike methods using task-specific fine-tuning^1,14,54^, semantic design requires no additional training that could bias generations towards characterized examples. Second, in contrast with approaches that specify function through natural language descriptions from existing knowledgebases^55^, semantic design accesses the functional diversity embedded within genomic sequences. This enables functional design that can leverage biological processes that are not yet characterized. For example, we generated antitoxins that suggest broader functional compatibility between diverse toxin–antitoxin systems (Fig. 2f) and an anti-CRISPR that maps to a protein family with a different putative function (Fig. 3i). Third, by leveraging genomic context as functional conditioning, semantic design does not require structural or mechanistic hypotheses; indeed, protein design pipelines that filter out low-confidence structure predictions^46,56^ would have removed many of our functional designs. Semantic design therefore represents a powerful orthogonal approach to current biological design strategies.

Semantic design could be particularly valuable for generating novel starting points for directed evolution or rational protein design, providing access to functional protein sequences beyond the constraints of characterized natural sequences^57^. Genomic conditioning is also useful when specifying functions such as anti-CRISPR activity that could be accomplished by many structures and mechanisms^40^. Of note, semantic design is not limited to Evo 1.5 and can leverage any language model trained on prokaryotic or phage genomes. Improvements in genomic language models, as well as a better understanding of prokaryotic gene synteny, should therefore directly translate to improvements in semantic design.

Traditional biological sequence discovery using guilt by association, which motivates many ideas in this study, is constrained to observed evolutionary diversity generated over billions of years. By contrast, semantic design enables the rapid generation of extensive sequence diversity for a biological system of interest. To facilitate broader accessibility of this new source of sequence material, we have reported SynGenome, a database of 120 billion base pairs of AI-generated genomic sequences, which we have made publicly available. This resource enables researchers, especially those without resources to conduct large-scale sampling from generative models, to find synthetic sequences related to their function of interest. This data could potentially contain new molecular tools and provide insights into protein function and evolution (Fig. 4i,j).

Although semantic design represents a new level of sequence novelty and functional improvement for generative genomics, several fundamental limitations and challenges remain. Autoregressive generation is prone to sampling repetitive sequences or to hallucinating realistic but non-functional designs. In addition, semantic design may yield genes that are contextually related to the prompt but encode unrelated functions: for instance, generating regulatory proteins controlling the expression of a gene with a desired function rather than the gene itself. Semantic design therefore requires both in silico filtering and experimental testing to validate downstream functions. Semantic design is also limited to functions encoded by contextual relationships in nature, particularly in prokaryotic organisms. However, we note that only a small fraction of prokaryotic functional diversity has been discovered and that the mining of this diversity has led to powerful biotechnologies such as PCR, optogenetics and genome editing^58–60^. Functional conditioning based on gene synteny also does not extend to many eukaryotic design tasks; however, future eukaryotic applications of semantic design could potentially leverage learned associations between proximal coding and non-coding DNA or within gene clusters.

Looking forward, the development of more capable pretrained models and an increase in sequencing data could reinforce the capabilities of semantic design. We also anticipate that combining the rich information learned by pretrained models with more advanced inference-time strategies will improve generation quality. Genomic language models that generate multi-component systems, as was done with our T2TA systems, could accelerate the development of synthetic biological circuits, pathways or even genomes. By leveraging semantic design, exploration of synthetic genomic space may reveal biological discoveries that complement and extend beyond those discovered in natural organisms.

## Methods

### Evo 1.5 pretraining

Evo 1.5 was generated by extending the pretraining of the Evo 1 model, originally trained at a sequence context of 8,192 tokens with an initial learning rate of 0.003 after a warmup of 1,200 iterations; a cosine decay schedule with a maximum decay iteration of 120,000 and a minimum learning rate of 0.0003. Training used a global batch size of 4,194,304 tokens and 75,000 iterations, processing a total of 315 billion tokens. Other details on hyperparameters related to the model architecture and optimizer can be found in ref. ^1^. Pretraining of the model, including all model states, optimizer states, data loading schedule and learning rate schedule, was resumed from 75,000 iterations to 112,000 iterations (processing a total of 470 billion tokens). The model trained up to 112,000 iterations is referred to as Evo 1.5.

As in Nguyen et al.^1^, Evo 1.5 was trained on the OpenGenome dataset, a comprehensive collection of prokaryotic genomic sequences. In brief, the dataset consists of approximately 80,000 prokaryotic genomes from bacteria and archaea and over 2 million phages and plasmid sequences, totalling approximately 300 billion nucleotides. OpenGenome was carefully curated to provide diverse, non-redundant genomic sequences from three primary sources: (1) bacterial and archaeal genomes from the Genome Taxonomy Database^61^, (2) prokaryotic viral sequences from the IMG/VR database^62^, and (3) plasmid sequences from the IMG/PR database^63^. To reduce redundancy, only representative genomes for each species, viral operational taxonomic unit, or plasmid taxonomic unit were retained. The dataset was further filtered to exclude potential eukaryotic viruses and sequences with poor taxonomic specificity. The training data include both positive and negative strands of the genomic sequences, allowing the model to learn the complementary nature of DNA. A detailed description of the dataset curation process is provided in Nguyen et al.^1^.

### Autoregressive sampling

To sample from Evo, a standard low-temperature autoregressive sampling algorithm was used. Sampling code (https://github.com/evo-design/evo/) based on the reference implementation (https://github.com/togethercomputer/stripedhyena) that leverages kv-caching of Transformer layers and the recurrent formulation of hyena layers^64,65^ was used to achieve efficient, low-memory autoregressive generation. Parameter optimization was performed across various temperatures (0.1–1.5, increments of 0.1), top-*k* values (1–4) and top-*p* values (0.1–1.0, increments of 0.1) using the Evo 1.5 model. For each parameter combination, 100 1,000-nt sequences were generated from a test set of five gene prompts encoding 50% of a highly conserved protein. Following identification of ORFs in generated sequences using Prodigal (v2.6.3, default parameters, -p meta) with default parameters in metagenome mode (-p meta)^66^, generated proteins were aligned against the full-length prompt protein sequence using MAFFT (v7.526)^67^ for sequence identity calculations. For evaluating sequence degeneracy, DustMasker (v2.14.1+galaxy2)^68–70^ was run across the full-length generations using default parameters and the proportion of masked nucleotides was calculated. Final parameters (temperature = 0.7, top-*k* = 4, top-*p* = 1.0) were selected based on maximizing sequence completion accuracy while maintaining DustMasker proportions below 0.2, a value chosen to be slightly higher than the typical frequency of non-coding DNA in prokaryotic genomes^71,72^. All code for sampling and downstream analysis using Evo was written in Python (v3.11.8).

### Sequence completion prompt compilation and evaluation

Sequences of highly conserved genes from across prokaryotic biology were downloaded in FASTA format from NCBI GenBank^73^. Selected genes included *rpoS* from *E. coli* K-12 (GenBank: NC_000913.3, coordinates c2866559–2867551), *gyrA* from *S. enterica* LT2 (GenBank: NC_003197.2, coordinates c2373710–2376422) and *ftsZ* from *H. volcanii* DS2 (GenBank: NC_013967.1, coordinates 643397–644536). Prompts were prepared by extracting 30%, 50% and 80% sequence lengths from the 5′ end. For the analysis of the gene completion ability of Evo 1.5 using sequences with varying levels of conservation, sequences encoding moderately conserved (*gloA* and *pilA*) and poorly conserved (*tnsA* and *yagL*) genes *gloA* from *E. coli* K-12 (GenBank: U57363.1, coordinates 1–408), *pilA* from *Pseudomonas aeruginosa* (GenBank: AE004091.2, coordinates c5069082–5069531), *tnsA* from *E. coli* O3 (GenBank: NZ_JALKIH010000010.1, coordinates 27218–28039) and *yagL* from *E. coli* K-12 (GenBank: U73857.1, coordinates c1018–1716) were used.

Sequence completion performance was evaluated across varying prompt lengths (30%, 50% and 80% of input sequence) using optimal sampling parameters for the Evo 1.5, Evo 1 8K (previous version of Evo trained with context length of 8,192 tokens) and Evo 1 131K (previous version of Evo trained with context length of 131,072 tokens) models (temperature = 0.7, top-*k* = 4, top-*p* = 1)^1^. For each prompt, 500 sequences of length 2,500 nt were generated and filtered to remove generations with DustMasker proportions above 0.2. Prompts were subsequently appended to the start of each generated sequence and ORFs were identified using Prodigal (v2.6.3, default parameters, -p meta) with default parameters in metagenome mode (-p meta). Generated proteins were then aligned against their corresponding natural sequences using MAFFT (v7.526) with default parameters for sequence identity calculations.

### Operon completion prompt compilation and evaluation

Sequences encoding the *trp* operon and *modABC* operon from *E. coli* K-12 (GenBank: NC_000913.3) were downloaded in FASTA format from NCBI GenBank. For *modABC*, prompts were prepared from the full coding sequences for *modA* (coordinates 795089–795862), *modB* (coordinates 795862–796551), *modC* (coordinates 796554–797612) and *acrZ* (coordinates 794773–794922). For *trp*, prompts were prepared from the full coding sequences for *trpE* (coordinates c1321384–1322946), *trpD* (coordinates c1319789–1321384), *trpC* (coordinates c1318427–1319788), *trpB* (coordinates c1317222–1318415) and *trpA* (coordinates c1316416–1317222). For testing the ability of the model to generate sequences on the antisense strand, the reverse complement of each sense strand-derived prompt sequence was generated using Biopython^74^.

Sequence completion performance was evaluated across the compiled operon completion prompts using previously identified optimal sampling parameters for Evo 1.5. For each prompt, 5,000 sequences of length 2,500 nt were generated. Following filtering of generations with DustMasker proportions above 0.2 and identification of ORFs using Prodigal (v2.6.3, default parameters, -p meta), directional completion was assessed by searching *trpE*-prompted generations for *trpD*-like ORFs, *trpD* reverse complement-prompted generations for *trpE*-like ORFs, and similar pairing combinations across both *modABC* and *trp* operons. Protein sequences were then aligned against their corresponding wild-type proteins for sequence identity calculations using MAFFT (v7.526). Structural similarity was evaluated by generating protein structure predictions using AlphaFold 3 (ref. ^75^) for both generated and wild-type sequences, with structural alignments and TM-score calculations performed using TM-align^76^. Natural and predicted protein structures were subsequently visualized using ChimeraX^77^.

### Positional entropy evaluation

Per-position amino acid and nucleotide entropies were calculated from multiple sequence alignments of 500 generated and natural *modB* and *trpA* sequences. Natural *modB* and *trpA* sequences were fetched by querying ‘ModB’ and ‘TrpA’ in NCBI protein, filtering by bacteria, and downloading the corresponding amino acid and nucleotide sequences in format ‘FASTA’ and ‘FASTA CDS’, respectively. Generated *modB* and *trpA* sequences were chosen by selecting a random sample of 500 *modB* and *trpA* ORFs (more than 80% sequence identity to *E. coli* sequence) from the *modB* and *trpA* sequences generated by prompting with *modA* and *trpB*, respectively, during the operon completion evaluation. First, nucleotide and amino acid sequences were aligned with MAFFT (v7.526) and trimmed to remove gaps appearing in more than 80% of sequences. For each position *i*, the Shannon entropy was then calculated as *H* = –Σ(*p*_*i*_ × log_2_(*p*_*i*_)) and normalized by dividing the calculated entropy by the maximum Shannon entropy (2 for nucleotides meaning all four bases are equally present, 4.32 for amino acids meaning all 20 standard amino acids are equally present), where *p*_*i*_ represents the frequency of each amino acid or nucleotide at that position.

### Analysis of species tag-prompting methods

For evaluation of the effect of species-specific prompting on sequence completion, sequences encoding *dnaK* (GenBank: NC_000913.3, coordinates 12163–14079) and *recA* (GenBank: U00096.3, coordinates c2822708–2823769) in *E. coli* K-12 and *secY* (GenBank: AF395886.1, coordinates 203–1669) and *tfb2* (GenBank: AF143693.1, coordinates 140–1138) in *H. volcanii* were downloaded in FASTA format from NCBI GenBank^73^. Base prompts were prepared by extracting 50% of the sequence lengths from the 5′ end. To generate species-specific prompt tags, the specific domain, phylum, class, family, genus, order and species information was extracted for each species from the Global Biodiversity Information Facility API (https://api.gbif.org/) and appended to the start of each base prompt. The species-specific prompts used are shown below:

1. |d__Bacteria;p__Pseudomonadota;c__Gammaproteobacteria;o__Enterobacterales;f__Enterobacteriaceae;g__*Escherichia*;s__*Escherichia* | |
2. |d__Archaea;p__Halobacteriota;c__Halobacteria;o__Haloferacales;f__Haloferacaceae;g__*Haloferax*;s__*Haloferax volcanii* | |

Sequence completion performance was evaluated by sampling the Evo 1 131K model using prompts with and without appended species tags following the method described in the section ‘Sequence completion prompt compilation and evaluation’ above.

To evaluate the effect of species-specific prompts on sequence entropy, prompts encoding *fusA* (GenBank: AH002539.2, coordinates 1243–1521) upstream of the evaluated *tufA* gene encoding EF-Tu from *E. coli* K-12 were fetched as described in the section ‘Operon completion prompt compilation and evaluation’. Sampling was performed as described before, but with species-specific prompt tags appended to the start of each prompt. Positional entropy was determined for *tufA* sequences generated with and without species tags as described in the section ‘Positional entropy evaluation’.

### Amino acid substitution analysis

Generated (see ‘Operon completion prompt compilation and evaluation’) and natural sequences encoding genes in the *trp* operon and *modABC* operon were first filtered to select for those with over 95% and less than 100% minimum amino acid sequence identity to their respective wild-type *E. coli* K-12 reference sequence using MMseqs2. From these filtered sequences, 100 sequences encoding each of *modA*,* modB*,* modC*, *acrZ*, *trpA*,* trpB*,* trpC*,* trpD* and* trpE* were randomly selected for both the Evo-generated and natural sequence groups. These sequences were subsequently aligned against their respective *E. coli* K-12 reference sequence using pairwise alignment with MAFFT (v7.526) and default parameters. Following alignment, amino acid substitutions were identified at each aligned position by comparing variant residues to the *E. coli* K-12 reference, excluding gap positions from analysis. Each substitution was then scored using the BLOSUM62 (ref. ^78^) matrix, with scores greater than or equal to 0 indicating biochemically conservative changes and scores less than 0 indicating non-conservative changes. BLOSUM scores for substitutions across all evaluated genes in the *trp* and *modABC* operons were then aggregated and plotted to get the final distribution of BLOSUM scores.

### T2TA prompt compilation and analysis

Genomic loci and sequences encoding T2TA system sequences were obtained by downloading the nucleotide sequence information for all experimentally validated T2TAs from the TADB 3.0 database^24^. Using the NCBI Entrez Programming Utilities API (EFetch from the nuccore database using the genomic loci from TADB 3.0)^73^, the 500 bp of upstream and downstream flanking sequence were extracted for each T2TA locus. In total, for each T2TA system, eight types of prompts were prepared: (1) individual toxin sequences, (2) individual antitoxin sequences, (3) the reverse complement of individual toxin sequences, (4) the reverse complement of individual antitoxin sequences, (5) the upstream context of the toxin loci, (6) the downstream context of the toxin loci, (7) the upstream context of the antitoxin loci, and (8) the downstream context of the antitoxin loci. Following successful identification of an Evo-generated toxin (see ‘Evaluation of types II and III toxin activity’ below), conjugate antitoxins were subsequently generated via prompting with the generated DNA sequence encoding the toxin.

To evaluate the frequency with which each prompt type generated toxin and antitoxin sequences, remaining protein sequences following sequence complexity and pDockQ filtering (see ‘T2TA sampling and filtering’) were evaluated using HMMER (v3.3.0) hmmscan (https://hmmer.org) against the Pfam-A database (v35.0)^79,80^. Generations with Pfam matches against known type II toxin or antitoxin-related families were counted as hits and mapped back to the prompt type used to generate the sequence, with the generation frequency calculated as the number of toxin or antitoxin hits divided by the total number of remaining generations for each prompt classification.

### T3TA prompt compilation and analysis

Genomic loci and sequences encoding T3TA system sequences were obtained by downloading the nucleotide sequence information for all experimentally validated and computationally predicted T3TAs from the TADB 3.0 database^24^. Using the NCBI Entrez Programming Utilities API (EFetch from the nuccore database using the genomic loci from TADB 3.0), the 1,000 bp of upstream and downstream flanking sequence were extracted for each T3TA locus. For each T3TA system, eight types of prompts were prepared: (1) individual toxin sequences, (2) individual antitoxin sequences, (3) the reverse complement of individual toxin sequences, (4) the reverse complement of individual antitoxin sequences, (5) the upstream context of the toxin loci, (6) the downstream context of the toxin loci, (7) the upstream context of the antitoxin loci, and (8) the downstream context of the antitoxin loci.

To evaluate the frequency with which each prompt type generated toxin and antitoxin sequences, generations with Rfam or Pfam matches against known T3TA-related families (see ‘T3TA sampling and filtering’) were mapped back to their prompt type and classified as hits. The overall generation frequency was calculated by dividing the number of hits by the total number of remaining generations for each prompt classification.

### T2TA sampling and filtering

To generate T2TA candidates, 53,104 sequences of 2,000 nucleotides each were first generated using Evo 1.5 (temperature = 0.7, top-*k* = 4, top-*p* = 1.0) from our compiled T2TA prompts. A multi-stage filtering pipeline was then applied to identify promising candidates. First, Prodigal (v2.6.3, default parameters, -p meta) was used to identify ORFs, excluding sequences containing proteins over 300 amino acids or less than to amino acids, resulting in 130,754 called proteins total^66^. Next, SegMasker (v2.14.1+galaxy2) with default parameters^69^ was used to remove sequences containing low-complexity regions with limited amino acid diversity, with 58,704 proteins remaining post-filtering. Next, any proteins that belonged to generations with only one passing protein were removed, resulting in 32,181 remaining protein candidates.

Protein–protein interaction potential of co-generated ORFs by co-folding all ORF pairs within each remaining generation was then assessed using ESMFold^17^. Generations were retained if they contained paired proteins with pDockQ^81^ scores greater than 0.23 and individual pLDDT scores greater than 0.3, resulting in 945 remaining pairs of proteins. Following the removal of any protein pairs with more than 40% sequence identity (MMseqs2^82^) between both proteins, 777 proteins remained. To identify novel candidates, the remaining sequences were searched against the non-redundant protein sequence database using BLAST^68^ (*e*-value cut-off of 0.05), selecting for generations containing at least one component with no significant BLAST hits to known toxins or antitoxins and the other component matching a known toxin or antitoxin. Following this filtering step, a total of 36 protein pairs remained. Ten final toxin candidates were then selected based on high-confidence interaction prediction using AlphaFold 3 (ref. ^75^).

Following the identification of functional toxin candidates via experimental testing, in which two toxins were found to be active, four were unable to be successfully cloned and three were inactive, the Evo-generated sequence encoding the strongest Evo-generated toxin, EvoRelE1, was used as a prompt to generate further diversified antitoxin candidates. After generating a total of 3,000 sequences from the EvoRelE1 prompt, 7,708 generated ORFs were filtered as above (744 remaining) before being co-folded with EvoRelE1. As with the first round of generations, candidates were filtered for high pDockQ scores, moderate pLDDT scores using ESMFold-derived co-folds (122 candidates remaining) and less than 40% identity to known antitoxins using BLAST (43 candidates remaining) before being evaluated for strong predicted co-folds using AlphaFold 3 (ipTM > 0.7). Remaining antitoxin candidates were further characterized using Foldseek Search Server^83^ searches of the AlphaFold 3-predicted structures (probability threshold of 0.6), blastp searches against the non-redundant protein database (*e*-value threshold of 1) and HHpred searches (probability threshold of more than 90%)^84^ to select a final of ten antitoxin candidates.

### T3TA sampling and filtering

To generate T3TA candidates, 25,960 sequences of 3,000 nucleotides each were first generated using Evo 1.5 (temperature = 0.7, top-*k* = 4, top-*p* = 1.0) from our compiled T3TA prompts. A multi-stage filtering pipeline was then applied to identify promising candidates. First, Prodigal (v2.6.3, default parameters, -p meta) was used to identify ORFs^66^, excluding sequences containing proteins over 400 amino acids or less than 50 amino acids, resulting in 80,298 called proteins total. Next, SegMasker (v2.14.1+galaxy2) with default parameters^69^ was used to remove sequences containing low-complexity regions with limited amino acid diversity, with 34,131 proteins remaining post-filtering. On sequences with at least one high-quality ORF present, ESMFold and Tandem Repeats Finder^85^ were used to identify generations with at least one ORFs with a pLDDT > 0.3 and at least one tandem repeat respectively. Tandem Repeat Finder was run using parameters: match = 2, mismatch = 7, delta = 7, PM = 80, PI = 10, minscore = 50, maxperiod = 2,000, resulting in 3,847 remaining generations. Consensus and full repeats were subsequently folded using ViennaRNA’s RNAfold^86,87^. Following filtering to remove any called tandem repeats with no hairpins, minimum free energies of more than −3.0 and without all four nucleotides present, a total of 428 sequences remained.

Remaining ORFs and identified tandem repeats from the filtered generations were subsequently evaluated using HMMER (v3.3.0) hmmscan against the Pfam-A database^80^ (v35.0) and rnascan^88^ against the Rfam database (v15.0)^89^, respectively. Tandem repeats were also run against the AbiF5_iter3.CM and the diverse_rna_xinsi.CM files from Zilberzwige-Tal et al.^90^ using Infernal’s cmscan^91^. As a point of comparison, natural T3TA sequences were also run against Pfam-A, Rfam and the AbiF-related covariance models. Generated sequences were retained if they had a match (*E* < 0.05) to a Pfam domain that overlapped with Pfam domains found in natural type III toxin sequences or if they had a match to a Rfam or AbiF family annotation found in natural type III antitoxin sequences.

To account for natural type III antitoxin sequences that did not have existing covariance models and sequence divergent-generated RNA sequences, an RNA secondary structure-based filtering on the generated sequences was also implemented. In brief, consensus repeat and full secondary structures of the generated sequences were compared with that of type III antitoxin sequences from TADB 3.0 and Zilberzwige-Tal et al.^90^. First, structures of generated tandem repeats were pre-filtered to exclude candidates with significantly different consensus repeat lengths and base-pairing ratio similarities, using a weighted average of length similarity (0.6 weight; a score of 1.0 indicates perfect match) and base-pairing ratio similarity (0.4 weight; a score of 1.0 indicates perfect match) against natural type III antitoxin sequences (retained sequences with scores ≥ 0.3). Remaining candidates underwent structural motif comparison using vectorized representations of extracted patterns including hairpins, stems, bulges and unpaired regions, with Jaccard similarity scoring applied to binary motif occurrence matrices (retained sequences with scores ≥ 0.4). Following motif extraction, final candidates were determined by calculating cosine similarity of the generated sequences against all natural type III antitoxins using ten-dimensional feature vectors encoding other RNA properties including base-pair counts, pairing ratios, average and maximum stem and loop lengths, number of stems, minimum free energy values, minimum free energy per nucleotide, and hairpin counts (retained sequences with similarities ≥ 0.7).

Sequences passing the Pfam, Rfam, AbiF CM, or RNA secondary structure filtering metrics were retained as final generations, for a total of 125 candidates. From this pool, a total of 36 toxin candidates and 10 antitoxin candidates were selected to experimentally validate, with selection criteria including sequence novelty for toxin candidates and IDT synthesizability for antitoxin candidates.

### Cloning of T2TA sequences

Sequences encoding Evo-generated T2TAs were codon optimized for *E. coli* expression using the codon optimization tool in IDT and synthesized as eBlocks. The toxin plasmid backbone was prepared by PCR amplification (New England Biosciences) and gel purification (Qiagen) of pAraSpCas9 + spMu^44^ to exclude the Cas9, CRISPR RNA, trans-activating CRISPR RNA and guide RNA sequences (Supplementary Data 1), creating an empty arabinose-inducible vector with spectinomycin resistance. Codon-optimized toxin sequences were subsequently inserted into the modified backbone using Gibson Assembly (New England Biosciences) according to the manufacturer’s recommendations.

For antitoxin cloning, the pZE21_tetR-AcrIIA4_kanR vector^44^ (Supplementary Data 1) was digested with EcoRI and HindIII (New England Biosciences) and gel purified (Qiagen) to remove the AcrIIA4 sequence. Codon-optimized antitoxin sequences were then inserted into the digested vector using Gibson Assembly (New England Biosciences).

Assembled plasmids were transformed into chemically competent Stellar *E. coli* HST08 cells (Takara Biosciences), and positive clones were selected on LB agar plates containing either 50 μg ml^−1^ spectinomycin (for the toxin constructs) or 50 μg ml^−1^ kanamycin (for the antitoxin constructs). Plasmid sequences were then confirmed by Sanger sequencing (Elim Biosciences).

### Cloning of T3TA sequences

Type III Evo-generated toxin candidates were codon optimized, synthesized and cloned following the methods outlined in ‘Cloning of T2TA sequences’.

For type III antitoxin cloning, sequences encoding the J23119(SpeI) promoter and a rho-independent terminator from the pAraSpCas9 + spMu plasmid were appended immediately upstream and downstream of the Evo-generated type III antitoxin sequences. The combined sequences were subsequently synthesized by IDT as eBlocks. For type III antitoxin cloning, the pZE21_tetR-AcrIIA4_kanR vector^44^ (Supplementary Data 1) was digested with MfeI and PvuI (New England Biosciences) and gel purified (Qiagen) to remove the AcrIIA4 and pLtetO-1 promoter sequences. Synthesized type III antitoxin sequences were then inserted into the digested vector using Gibson Assembly (New England Biosciences).

Assembled plasmids were transformed into chemically competent Stellar *E. coli* HST08 cells (Takara Biosciences), and positive clones were selected on LB agar plates containing either 50 μg ml^−1^ spectinomycin (for the toxin constructs) or 50 μg ml^−1^ kanamycin (for the antitoxin constructs). Plasmid sequences were then confirmed by Sanger sequencing (Quintara Biosciences).

### Evaluation of types II and III toxin activity

Toxin activity was assessed through growth inhibition assays in *E. coli* NEB Turbo cells (New England Biosciences) for type II toxin candidates and Stellar *E. coli* HST08 (Takara Biosciences) for type III toxin candidates. Cells transformed with toxin constructs were first grown overnight in LB medium containing 50 μg ml^−1^ spectinomycin. Cultures were then diluted 1:10 into 1 ml fresh LB medium supplemented with 50 μg ml^−1^ spectinomycin and 2 mg ml^−1^ arabinose to induce toxin expression. After 2 h of induction, cultures were further diluted 1:40 into 200 μl of the same medium in triplicate wells of a 96-well plate. Growth was monitored using a Tecan Spark plate reader at 37 °C with orbital shaking, measuring an optical density at 600 nm (OD_600_) at 30-min intervals over a 12 h period. For each toxin, an uninduced control grown in arabinose-free LB–spectinomycin media was used. Growth curves were analysed to evaluate the extent of toxin-mediated growth inhibition. To calculate relative survival, the final OD_600_ measurement following the 12 h growth period for each toxin was divided by the final OD_600_ value of its respective uninduced control. For comparisons between EvoT1 and natural ToxN, statistical comparisons were done using a one-sided Student’s *t*-test against the induced ToxN negative control, with the *P* value for EvoT1 being 0.1105.

### Evaluation of type II antitoxin activity

For antitoxin evaluation, *E. coli* NEB Turbo cells were co-transformed with both toxin and antitoxin constructs (1:2 ratio of toxin to antitoxin) and grown overnight in LB medium containing 50 μg ml^−1^ spectinomycin and 50 μg ml^−1^ kanamycin. Cultures were then diluted 1:10 into 1 ml fresh LB medium supplemented with both antibiotics and 2 mg ml^−1^ arabinose to induce toxin expression. After 2 h of induction, cultures were further diluted 1:40 into 200 μl of the same medium in triplicate wells of a 96-well plate per condition. For each antitoxin, an uninduced control grown in arabinose-free LB–spectinomycin + kanamycin media was used. Growth was monitored using a Tecan Spark plate reader at 37 °C with orbital shaking, measuring OD_600_ at 30-min intervals over a 12 h period. Growth curves were analysed to evaluate antitoxin-mediated rescue of growth. To calculate relative survival, the final OD_600_ measurement following the 12 h growth period for each toxin–antitoxin pair was divided by the final OD_600_ value of its respective uninduced control. Statistical comparisons were done using a one-sided Student’s *t*-test against the EvoRelE1 + eGFP negative control, with *P* values for EvoAT1–4 being 1.61 × 10^−7^, 1.42 × 10^−7^, 2.30 × 10^−5^ and 1.69 × 10^−5^, respectively.

### Evaluation of type III antitoxin activity

For type III antitoxin evaluation, Stellar *E. coli* HST08 cells (Takara Biosciences) were co-transformed with both toxin and antitoxin constructs (1:3 ratio of toxin to antitoxin) and grown overnight in LB medium containing 50 μg ml^−1^ spectinomycin and 50 μg ml^−1^ kanamycin. Cultures were then diluted 1:10 into 1 ml fresh LB medium supplemented with both antibiotics and 2 mg ml^−1^ arabinose to induce toxin expression. After 3 h of induction, cultures were further diluted 1:40 into 200 μl of the same medium in triplicate wells of a 96-well plate per condition. For each antitoxin, an uninduced control grown in arabinose-free LB–spectinomycin + kanamycin media was used. Growth was monitored using a Tecan Spark plate reader at 37 °C with orbital shaking, measuring OD_600_ at 15-min intervals over a 12 h period. Growth curves were analysed to evaluate antitoxin-mediated rescue of growth. To calculate relative survival, the final OD_600_ measurement following the 12 h growth period for each toxin–antitoxin pair was divided by the final OD_600_ value of its respective uninduced control. Statistical comparisons were done using a one-sided Student’s *t*-test against the induced ToxN negative control, with the *P* value for EvoAT6 being 1.84 × 10^−8^.

### Anti-CRISPR prompt compilation and analysis

Genomic sequences containing known type II Cas9-targeting anti-CRISPR (*acr*) genes and their associated operons were obtained from previously characterized Acr systems annotated in AcrDB^36^. Using the Entrez API, Acr coding sequences along with 500 bp of flanking sequence both upstream and downstream of each *acr* locus were extracted. For each Acr system, six types of prompts were prepared: (1) individual Acr sequences, (2) Acr-associated (*aca*) gene sequences, (3) the reverse complement of individual Acr sequences, (4) the reverse complement of Acr-associated (*aca*) gene sequences, (5) the upstream context of the Acr loci, and the (6) the downstream context of the *acr* loci.

To evaluate the frequency with which each prompt type generated likely anti-CRISPR sequences, remaining generations following PaCRISPR filtering (see ‘Acr sampling and filtering’) were mapped back to their prompt type and classified as hits. The overall generation frequency was calculated by dividing the number of hits for each prompt type by the total number of remaining generations of that prompt classification.

### Anti-CRISPR sampling and filtering

To generate Acr candidates, a total of 3,160 sequences of 1,000 nucleotides each were sampled using Evo 1.5 (temperature = 0.7, top-*k* = 4, top-*p* = 1.0) from our compiled Acr prompts. A multi-stage filtering pipeline was then applied to identify promising candidates. First, Prodigal (v2.6.3, default parameters, -p meta) was used to identify ORFs, excluding sequences containing proteins over 200 amino acids or less than 50 amino acids long, resulting in a total of 4,223 potential ORFs. Next, SegMasker (v2.14.1+galaxy2, default parameters) was used to remove sequences containing over 50% low-complexity regions or limited amino acid diversity, filtering the generations down to 1,391 candidates. Sequences were subsequently folded using ESMFold to remove any candidates with very low-confidence folds (pLDDT < 0.25) or no secondary structure, reducing the total candidates to 468. Candidate sequences were then evaluated using PaCRISPR, a machine learning model trained to identify potential Acr proteins based on sequence features^43^. Following guidelines established by PaCRISPR, candidates scoring above 0.75 were considered potential Acrs and advanced to the next step, resulting in 131 remaining generations. Generated sequences classified as potential Acrs by PaCRISPR were then searched against the non-redundant protein sequence database using blastp (*e*-value cut-off of 1) to identify candidates with varying degrees of sequence novelty. From this pool of 131 candidates, 84 were selected for further screening based on sequence novelty, similarity to other screening candidates and PaCRISPR scores. For comparison, this filtration pipeline was also applied to evaluate sequences generated by prompting with randomly generated DNA sequences and non-Acr-related genomic sequences. The sequence diversity among the predicted Acrs was assessed by performing pairwise alignments using MAFFT (v7.526) on a set of 56 randomly selected sequences that scored above 0.75 in PaCRISPR. The resulting pairwise sequence identities were visualized using a matplotlib heatmap.

### Cloning of anti-CRISPR sequences

Sequences encoding Evo-generated Acr proteins were codon optimized for *E. coli* expression using the codon optimization tool in IDT and synthesized as eBlocks. To generate the cloning backbone, the pZE21_tetR-AcrIIA4-Coli_kanR vector (Supplementary Data 1) was digested with EcoRI and HindIII (New England Biosciences) to remove the AcrIIA4 sequence and gel purified (Qiagen). Codon-optimized Acr sequences were then inserted into the digested vector using Gibson Assembly (New England Biosciences) according to the manufacturer’s recommendations. Assembled plasmids were then transformed into chemically competent Stellar *E. coli* HST08 cells (Takara Biosciences), and positive clones were selected on LB agar plates containing 50 μg ml^−1^ kanamycin. Plasmid sequences were confirmed by Sanger sequencing (Elim Biosciences).

### Liquid culture assay for measuring anti-CRISPR activity

Acr activity was assessed through protection assays against SpCas9-mediated DNA cleavage in *E. coli*. NEB Turbo cells were first co-transformed with both the Acr expression plasmid and the CRISPR-targeting plasmid containing SpCas9 and a KanR-targeting guide RNA^44^ (pAraCas9 + Sp2 + Sp6 + I-SceI; Supplementary Data 1) and grown overnight in LB medium containing both 50 g ml^−1^ kanamycin and spectinomycin.

For liquid culture survival assays, 30 μl of overnight culture was diluted into 1 ml fresh LB medium containing spectinomycin and 0.2 mg ml^−1^ arabinose to induce SpCas9 expression and deplete the kanamycin resistance plasmid. After 7 h of growth, cultures were normalized to equal optical density and diluted 1:40 into 200 μl fresh LB medium containing 50 μg ml^−1^ kanamycin and spectinomycin in a 96-well plate to select for cells with active Acr proteins. Growth was monitored using a Tecan Spark plate reader at 37 °C with orbital shaking, measuring OD_600_ at 30-min intervals over an 8 h period. For each Acr, an uninduced control grown in arabinose-free LB–spectinomycin + kanamycin media was used to normalize growth values. Relative survival values were calculated by dividing the final OD_600_ measurement for each Acr following the 8 h growth period by the final OD_600_ value of its respective uninduced control.

Statistical comparisons were done using a one-sided Student’s *t*-test against the random sequence negative control and AcrIIA2, with *P* values for EvoAcr1–5 being 8.31 × 10^−6^, 6.19 × 10^−6^, 8.19 × 10^−6^, 1.43 × 10^−6^ and 8.18 × 10^−7^ against the negative control, respectively, and the *P* value for EvoAcr5 against AcrIIA2 being 5.07 × 10^−4^.

### Phage plaque assay for measuring anti-CRISPR activity

For phage protection assays, *E. coli* NEB Turbo cells were co-transformed with both the Acr expression plasmid and a modified SpCas9 plasmid containing a guide RNA targeting T4(GT7) phage (SpCas9-mrh2; Supplementary Data 1) at a 5:1 Acr:Cas9 ratio. Co-transformed cells were grown overnight in LB medium containing 50 μg ml^−1^ kanamycin and spectinomycin. Cultures were then diluted 1:10 into fresh LB medium supplemented with spectinomycin, kanamycin and 0.2 mg ml^−1^ arabinose to induce Cas9 expression. When cultures reached an OD_600_ of 0.4, 300 μl was mixed into 10 ml of 0.7% soft agar containing 50 μg ml^−1^ kanamycin, 50 μg ml^−1^ spectinomycin and 0.02 mg ml^−1^ arabinose. Plates were allowed to harden, and T4(GT7) phage at a titre of 1.7 × 10^8^ PFU ml^−1^ was serially diluted 1:5 and spotted onto the bacterial lawn. Plates were then incubated overnight at 37 °C before being imaged to visualize plaque formation.

### Measurement of Evo-generated sequence diversity

Sequence and structural diversity of generated toxin–antitoxin pairs and Acrs were assessed through a combination of sequence and structure-based searches. Protein sequences were searched against the NCBI non-redundant protein database using blastp under default parameters (*e*-value threshold of 1.0) to identify similar natural sequences. Overall sequence identity was defined as the percent identity of a given BLAST match multiplied by the query cover of the alignment. RNA sequences were searched against the NCBI non-redundant nucleotide database using blastn under default parameters (*e*-value threshold of 1.0). Reported BLAST results are from a run conducted on 14 July 2025. An additional search against OpenGenome was performed using MMseqs2 (v15.6f452) with maximum sensitivity (-s 7) and other parameters set to the default values^82^ to evaluate similarity to sequences in the training data. For more-sensitive HMM-based characterization of generated genes, all Evo-generated proteins were searched against PDB_mmCIF70_25_May using the HHpred^84,92^ webserver (https://toolkit.tuebingen.mpg.de/tools/hhpred), with probabilities greater than 90 or *E* less than 0.05 being deemed significant. Foldseek-based structural similarity was evaluated by searching AlphaFold 3-predicted structures against the AFDB-Swissprot and AFDB50 protein databases^93,94^ using the Foldseek Search Server (https://search.Foldseek.com/search)^83^, with values greater than 0.5 reported as significant hits following Foldseek guidelines^95^. Dali-based structural similarity was evaluated by searching AlphaFold 3-predicted structures against PDB100 and the AF-DB V2 *E. coli* subset using the Dali webserver with hits with *Z* > ((*n*/10) − 4) (*n* = residues in query structure) being labelled as strong significant matches following Dali guidelines (http://ekhidna2.biocenter.helsinki.fi/dali/)^49,96^. For each generated protein, the closest sequence and structural matches were identified from all searches (BLAST, MMseqs2, HHpred, Dali and Foldseek) and were evaluated to determine the degree of novelty compared with known proteins. Pairwise sequence identities were calculated from BLAST alignments, broader sequence similarity was calculated using HHpred probabilities and *e*-values, and structural similarities were quantified Foldseek TM-scores and Dali *z*-scores. For RNA HMM-based sequence similarity evaluation, generated type III antitoxins were searched against Rfam using rnascan^87,89^ using an *e*-value threshold of 0.05. For structural evaluation of generated RNA and natural RNA antitoxins, individual secondary structure predictions for full and consensus repeat sequences were obtained using ViennaRNA’s RNAfold^87^. Secondary structure similarity for the consensus repeats in EvoAT6 and Rfam ToxI were calculated using LocARNA^97^. The results of all these analyses can be found in Supplementary Data 2–5.

For the per-residue compositionality analyses (Extended Data Figs. 3a, 4l and 6a), the top 1,000 BLAST hits (*E* < 1.0) and MMseqs2 hits for each protein were compiled using the parameters described above, with the inclusion of an MMseqs2 run against TADB 3.0 (ref. ^24^) for toxin–antitoxin generations. Residue-level assignments were first determined by exact amino acid matching within the compiled BLAST and MMseqs2 results, with each query position assigned to the best-scoring hit. To avoid self-hits and confounding high-similarity alignments, matches with more than 95% sequence identity were excluded. Any unassigned regions were then integrated with a *k*-mer-based sequence matching algorithm to improve sequence coverage. To construct the *k*-mer database, all overlapping *k*-mers (lengths of 3–12 amino acids) were extracted from a random sample of 100,000 UniRef30 proteins shorter than 800 amino acids. For each unassigned region, the optimal set of non-overlapping exact *k*-mer matches that maximized sequence coverage while minimizing the number of distinct source proteins was identified. In brief, all possible exact *k*-mer matches for each unassigned position were enumerated and scored based on *k*-mer length, with longer matches receiving higher scores. These matches were treated as intervals, and an optimization algorithm was used to select the highest-scoring, non-overlapping subset. To encourage coherent assignments and minimize the number of proteins used, additional weighting was applied to favour proteins contributing multiple *k*-mers across all unassigned regions.

### SynGenome prompt compilation

Protein sequences from prokaryotic and phage organisms were retrieved from UniProt^98^, with all Swiss-Prot proteins with associated coding regions and a random sample of TrEMBL proteins with associated coding regions being used as starting points for prompts. Genomic contexts were retrieved via NCBI’s Entrez Programming Utilities (E-utilities) API, specifically using EFetch with the nuccore database using the coding sequence (CDS) annotations associated with the Protein Sequence accession in UniProt^73,98^. For the genomic identifier associated with each protein, three API calls were constructed: one to extract the coding sequence using sequence feature coordinates and two to extract the flanking regions by calculating positions 500 nucleotides upstream and downstream of the CDS boundaries. For CDS regions that were longer than 500 nucleotides, the prompt was derived from the final 500 nucleotides in the coding sequence. For CDS regions that were shorter than 500 nucleotides, the CDS sequence was trimmed to the nearest 100 bp. In addition, the reverse complement sequences for each region were generated using the Biopython Seq module, resulting in six distinct prompts per protein: upstream, CDS, downstream and their respective reverse complements. Associated functional annotations including Gene Ontology terms^47^, species names and InterPro domains^48^ were retrieved from REST API in UniProt for each protein using their UniProt accession numbers and linked to their corresponding prompts.

### SynGenome sampling

Sequences were generated using Evo 1.5 with optimized sampling parameters (temperature = 0.7, top-*k* = 4, top-*p* = 1.0). For each prompt, two sequences of 5,000 nucleotides in length were generated, yielding a total database size of over 120 billion base pairs. Generation was performed in parallel across multiple compute nodes to facilitate large-scale sequence production.

### Data filtering of SynGenome sequences

Generated sequences underwent a multi-step filtering pipeline to remove low-complexity regions while preserving biologically plausible features. Initial validation removed any invalid characters from the nucleotide sequences. These sequences were then processed using DustMasker (NCBI BLAST+ v2.16.0) with a masking level of 30 (-level 30) and FASTA output format (-outfmt fasta) to identify low-complexity regions. Following DustMasker processing, two additional filtering steps were implemented: removal of successive 100 nucleotide chunks from the sequence end if they contained more than 40% masked bases, and elimination of any continuous masked regions longer than 800 nucleotides. Sequences were excluded from the final database based on several criteria: length below 100 nucleotides, masked base content exceeding 80% in sequences shorter than 2,000 nucleotides, complete masking of all bases or empty/NaN values. The sequences passing these filtering criteria were retained in the final database.

### Prediction of SynGenome ORFs

ORFs were identified in the filtered sequences using Prodigal (v2.6.3, default parameters, -p meta) with default parameters and metagenomic prediction mode (-p meta). Predictions were initially refined by excluding sequences shorter than 40 amino acids or longer than 1,200 amino acids and sequences with incomplete protein sequences. Following basic filtration, low-complexity regions were identified using SegMasker (v2.14.1+galaxy2, window size of 15, locut of 1.8, hicut of 3.4), with sequences containing more than 20% masked regions being excluded. Additional complexity filters removed sequences with fewer than 12 unique amino acids or highly repetitive *k*-mer patterns (*k* = 3–10, threshold > 40% coverage). This multi-step filtering process ensured the retention of higher-quality protein predictions while removing potentially spurious or low-complexity sequences.

### Creation of the SynGenome domain association network

Protein family domains in filtered protein predictions from SynGenome (see ‘Prediction of SynGenome ORFs’) were identified using HMMER (v3.3.0) hmmscan against the Pfam-A database^80^ (v35.0) with default parameters (*e*-value cut-off of 0.1). Next, to analyse domain annotation patterns across SynGenome, a directed graph connecting Pfam domains found in prompt sequences to those found in generated response sequences (nodes) was constructed, with edge weights representing co-occurrence frequencies. The resultant network was visualized using Pyvis^99^, with the top 10,000 strongest edges being visualized. To ensure that no single highly connected node dominated the network, each node was restricted to a maximum of five top edges.

To identify connections between Pfam clans, Pfam domains in both prompt and response sequences were mapped back to their corresponding clan using metadata in Pfam-A (v35.0). As before, a network connecting Pfam clans found in prompts to those found in responses was created, with nodes representing individual Pfam clans and edge weights corresponding to co-occurrence frequencies in prompt–response pairs. Statistics on the network were calculated using igraph (v0.11.6)^100^.

### Evaluation of SynGenome DUF associations

To identify enriched DUF associations from the SynGenome domain association network (see ‘Creation of the SynGenome domain association network’), first, a series of DUFs present in the top 500,000 most connected nodes were identified. These nodes were then analysed for their associations within the network. For each domain pair involving at least one of these identified DUFs, the co-occurrence frequency, representing the observed edge weight, was directly determined from the network. Next, the relative enrichment of each DUF-connected edge was calculated by dividing the observed edge weight by the expected weight. This expected weight was calculated as the product of the sums of incident edge weights of the two connected domains, normalized by the total weight of the entire network. Co-occurrence frequency was then plotted against relative enrichment to identify domains exhibiting both high co-occurrence rates and strong association strength with each DUF.

### Identification of structurally chimeric proteins in SynGenome

To identify structurally chimeric proteins among the proteins generated in SynGenome, first, all proteins in SynGenome that had significant Pfam hits (see ‘Creation of the SynGenome domain association network’) were folded using ESMFold^17^, resulting in approximately 3.7 million predicted structures. Sequences were then filtered for those that had pLDDT scores greater than 0.4 and clustered at 90% sequence identity using MMseqs2 (ref. ^82^), with a representative sequence being chosen from each cluster. Predicted structures of the remaining sequences were then sharded into individual batches of 100,000 proteins and turned into Foldseek databases^83^. Each database was subsequently aligned against itself and all other shard databases using Foldseek (v10-941cd33; *e*-value of 1, sensitivity of 7 and maximum sequences of 300), effectively resulting in a structural all-by-all alignment of SynGenome proteins against each other. The resultant alignments were then filtered to exclude alignments with TM-scores less than 0.3 and turned into a DuckDB for simplified querying. To rapidly identify proteins that appeared to be chimeras of other unrelated SynGenome proteins, 1% of query proteins with moderate structural connectivity (2–10 Foldseek hits) to other SynGenome proteins were randomly sampled as candidate chimeras. For each potential chimera, all pairwise combinations of its corresponding target proteins were enumerated as candidate parent pairs. These parent pairs were then filtered to remove any pairs that showed direct structural similarity to each other, retaining only those where both proteins were structurally similar to the chimera candidate protein but not to each other.

To find chimera candidates containing potential domain fusions, candidates were further filtered to retain groups where the chimera candidate contained domains from both parents while the parents had no overlapping domains. The resulting chimera candidates were then queried against the NCBI non-redundant protein database (*E* < 0.05), with proteins with greater than 70% sequence identity to any single database hit being removed. Domain architectures of the resultant chimeras were also cross-referenced against InterPro to determine chimera candidates that appeared to be novel domain fusions. This yielded a final set of structurally chimeric proteins with limited sequence identity to any one protein.

### Creation of SynGenome website

The SynGenome website was implemented with HTML, CSS and JavaScript to provide a searchable web interface (https://evodesign.org/syngenome/). The interface allows users to query sequences using protein names, UniProt IDs, InterPro domains, species names or Gene Ontology terms. The database was structured to maintain associations between generated sequences and their corresponding prompt annotations. The website also includes the SynGenome domain association network (see ‘Creation of the SynGenome domain association network’), which can be queried using Pfam IDs, clans and keywords, and displays the 10,000 strongest prompt–response domain connections across SynGenome (https://evodesign.org/syngenome/network). The raw SynGenome data and ESMFold-predicted structures of generated proteins with significant Pfam hits are hosted on Hugging Face for public access (https://huggingface.co/datasets/evo-design/syngenome-protein-structures/tree/main and https://huggingface.co/datasets/evo-design/syngenome-uniprot).

### Creation of SynGenome UMAP and Leiden clusters

To generate the UMAP, first, a random sample of 50,000 sequences encoding at least one ORF with prompts derived from the CDS was extracted from SynGenome. This random sample was subsequently filtered to remove generations with less than 40% or more than 60% GC content, resulting in a final set of 36,762 generations. Embeddings were generated for both the prompt and corresponding generated sequences by extracting activations from the MLP layer 3 in the 6th hyena block of Evo 1.5’s architecture before being mean pooled along the sequence dimension to create fixed-length representations. These high-dimensional embeddings were subsequently reduced to two dimensions using UMAP (*n*_neighbours = 15, min_dist = 0.1). Sequences were coloured according to their percent identity to sequences in the training data, calculated using MMseqs2 against a database of prokaryotic genomes used in model training. These high-dimensional embeddings were reduced to two dimensions using UMAP with ScanPy (v1.10.3)^101^ default parameters. Sequences were coloured according to their percent identity to sequences in the training data, calculated using MMseqs2 against OpenGenome. Graph-based clustering was performed using the default Leiden algorithm implemented in Scanpy. The resolution parameter was optimized by evaluating cluster stability across resolutions from 0.1 to 0.5, measuring both the number of clusters and the coefficient of variation of cluster sizes. A final resolution of 0.2 was selected for clustering based on these metrics.

### Comparison of SynGenome to natural prokaryotic sequences

To evaluate how representative SynGenome proteins were compared with natural prokaryotic sequences, first, a random sample of 36,762 5,000-nt sequences was taken from OpenGenome to match the number of sequences used in the representative sample of SynGenome (see ‘Creation of SynGenome UMAP and Leiden Clusters’). Following the procedure used for prediction of ORFs in SynGenome (see ‘Prediction of SynGenome ORFs’), Prodigal (v2.6.3, default parameters, -p meta) was used to identify potential ORFs in the sampled sequences and minimal filtering with SegMasker was applied to remove incorrectly called sequences. Length distributions of predicted SynGenome ORFs were compared with those from the random OpenGenome sample. Codon usage bias of the prompts and SynGenome generations were analysed by calculating the total frequency of each codon across all prompt ORFs and all generated ORFs and normalizing the frequencies to the total number of codons per dataset. Protein family domains were identified in both datasets using HMMER (v3.3.0) hmmscan against the Pfam-A database (v35.0)^80^ with default parameters (*e*-value cut-off of 0.01). Domain frequencies were compared between datasets using Fisher’s exact test with Benjamini–Hochberg multiple testing correction (minimum occurrence threshold of ten domains in both datasets). Domain frequency relationships were visualized using a log-scale scatterplot, with points coloured by absolute log_2_ fold change between datasets. Correlation between domain frequencies was assessed using the Pearson correlation coefficient calculated using the pearsonr function in SciPy (v1.11.4)^102^. The distribution of domain occurrence frequencies was analysed by binning domains found in SynGenome and OpenGenome proteins into log-scale bins (2^*n*^ occurrences) and visualizing the count of unique domains per frequency bin for each.

To evaluate the associations captured by the SynGenome domain association network compared with natural prokaryotic syntenic associations, first, a set of 15 representative bacterial and archaeal genomes and their corresponding proteins were downloaded from the NCBI. These included *E. coli* K-12 MG1655 (GCF_000005845.2), *Bacillus subtilis* subsp. *subtilis* str. 168 (GCF_000009045.1), *Mycobacterium tuberculosis* H37Rv (GCF_000195955.2), *P. aeruginosa* PAO1 (GCF_000006765.1), *Staphylococcus aureus* NCTC 8325 (GCF_000013425.1), *Cyanobacterium aponinum* PCC 10605 (GCF_000317675.1), *Clostridium acetobutylicum* ATCC 824 (GCF_000008765.1), *Mycoplasma pneumoniae* M129 (GCF_910574535.1), *Helicobacter pylori 26695* (GCF_000307795.1), *Wolbachia pipientis* wMel (GCF_016584425.1), *Methanocaldococcus jannaschii* DSM 2661 (GCF_000091665.1), *H. volcanii* DS2 (GCF_000025685.1), *Sulfolobus acidocaldarius* DSM 639 (GCF_000012285.1), *Pyrococcus furiosus* DSM 3638 (GCF_008245085.1) and *Nitrosopumilus maritimus* SCM1 (GCF_000018465.1). All proteins from these genomes were annotated using HMMER (v3.3.0) hmmscan against the Pfam-A database (v35.0)^80^ and mapped back to their corresponding Pfam clan. To characterize natural genomic co-occurrence in a manner reminiscent of the generation methods in SynGenome, co-occurring genes were defined as those found within 5,000 bp of each other, with domain associations added between all Pfam clan hits from genes meeting this criterion. Each time a given Pfam clan association was observed, the weight of the edge connecting the domains was incremented, akin to the SynGenome network construction process (see ‘Creation of the SynGenome domain association network’). To account for the much larger number of proteins and associations in SynGenome, *z*-score normalization was applied to the Pfam clan edge weights in both SynGenome and natural genomes. *z*-scores were calculated as *z* = (*x* − *μ*)/*σ*, where *x* represents the original edge weight, *μ* is the mean edge weight and *σ* is the standard deviation of all edge weights within the SynGenome or natural genome co-occurrence network. *z*-scores for shared associations were subsequently plotted against each other for all shared Pfam clan associations occurring in frequencies of more than 10.

### Reporting summary

Further information on research design is available in the Nature Portfolio Reporting Summary linked to this article.

## Online content

Any methods, additional references, Nature Portfolio reporting summaries, source data, extended data, supplementary information, acknowledgements, peer review information; details of author contributions and competing interests; and statements of data and code availability are available at 10.1038/s41586-025-09749-7.

## Supplementary information

Supplementary Figures This file contains Supplementary Figures 1 and 2. Supplementary Figure 1: Genomic context of closest sequence matches of EvoAT1-4 and EvoAcr3; Supplementary Figure 2: Uncropped phage plaque images for Acr protection assay.

Reporting Summary

Supplementary Data 1 Prompts and DNA sequences for tested anti-CRISPR and toxin-antitoxin candidates. (1) Prompts and generated sequences for EvoRelE1 and EvoAT1-4. (2) Prompts and generated sequences for EvoT1 and EvoAT6. (3) Prompts and generations for EvoAcr1-5. (4) Prompts used for type II toxin generation. (5) Prompts used for type II antitoxin generation. (6) Prompts used for type III toxin and antitoxin generation. (7) eBlock sequences for all experimentally screened type II toxins and antitoxins. (8) eBlock sequences for all experimentally screened type III toxins and antitoxins. (9) eBlock sequences for all experimentally screened Acrs. (10) Primer and plasmid sequences used for cloning toxin-antitoxin constructs and Acr constructs.

Supplementary Data 2 Compiled Foldseek results for EvoRelE1, EvoAT1-4, EvoT1, and EvoAcr1-5. (1) Results for EvoAT1. (2) Results for EvoAT2. (3) Results for EvoAT3. (4) Results for EvoAT4. (5) Results for EvoRelE1. (6) Results for EvoT1. (7) Results for EvoAcr1. (8) Results for EvoAcr2. (9) Results for EvoAcr3. (10) Results for EvoAcr4. (11) Results for EvoAcr5.

Supplementary Data 3 Compiled HHpred results for EvoRelE1, EvoAT1-4, EvoT1, and EvoAcr1-5. (1) Results for EvoAT1. (2) Results for EvoAT2. (3) Results for EvoAT3. (4) Results for EvoAT4. (5) Results for EvoRelE1. (6) Results for EvoT1. (7) Results for EvoAcr1. (8) Results for EvoAcr2. (9) Results for EvoAcr3. (10) Results for EvoAcr4. (11) Results for EvoAcr5.

Supplementary Data 4 Compiled Dali results for EvoRelE1, EvoAT1-4, EvoT1, and EvoAcr1-5. (1) Results summary for compiled hits, including calculated significance thresholds for each design. (2-6) Results for EvoAcr1 (2), EvoAcr2 (3), EvoAcr3 (4), EvoAcr4 (5), and EvoAcr5 (6) against PDB. (7-11) Results for EvoAcr1 (7), EvoAcr2 (8), EvoAcr3 (9), EvoAcr4 (10), and EvoAcr5 (11) against AFDB *E. coli*. (12-17) Results for EvoRelE1 (12), EvoAT1 (13), EvoAT2 (14), EvoAT3 (15), EvoAT4 (16), and EvoT1 (17) against PDB. (18-23) Results for EvoRelE1 (18), EvoAT1 (19), EvoAT2 (20), EvoAT3 (21), EvoAT4 (22), and EvoT1 (23) against AFDB *E. coli*.

Supplementary Data 5 Compiled BLAST results for EvoRelE1, EvoAT1-4, EvoT1, and EvoAcr1-5. (1) Results for EvoAT1. (2) Results for EvoAT2. (3) Results for EvoAT3. (4) Results for EvoAT4. (5) Results for EvoRelE1. (6) Results for EvoT1. (7) Results for EvoAcr1. (8) Results for EvoAcr2. (9) Results for EvoAcr3. (10) Results for EvoAcr4. (11) Results for EvoAcr5.

## Data Availability

SynGenome is explorable and searchable (https://evodesign.org/syngenome/). Raw data have been deposited to Hugging Face datasets (https://huggingface.co/datasets/evo-design/syngenome-uniprot). Protein structures predicted by ESMFold for approximately 3.7 million genes in SynGenome have been deposited to Hugging Face datasets (https://huggingface.co/datasets/evo-design/syngenome-protein-structures). DNA, RNA and protein sequences used during our validation experiments are available in Supplementary Data 1. Data from our bioinformatic analyses of our generated sequences can be found in Supplementary Data 2–5. Sequence prompts for gene and operon completion evaluations, anti-CRISPR generation, toxin–antitoxin generation and SynGenome generation were derived from NCBI GenBank (https://www.ncbi.nlm.nih.gov/genbank/), AcrDB (https://bcb.unl.edu/AcrDB/), TADB 3.0 (https://bioinfo-mml.sjtu.edu.cn/TADB3/index.php) and Uniprot (https://www.uniprot.org/), as described in Methods. OpenGenome is openly available on Hugging Face (https://huggingface.co/datasets/LongSafari/open-genome). All newly created materials are available on reasonable request to the corresponding author.

## Code availability

The code and documentation for generation with Evo 1.5 based on a user-defined prompt (https://github.com/evo-design/evo/) and the scripts for semantic design of the toxin–antitoxin systems and anti-CRISPRs (https://github.com/evo-design/evo/tree/main/semantic_design) are available on GitHub. The Evo 1.5 model is available under an open-source license at Hugging Face (https://huggingface.co/evo-design/evo-1.5-8k-base).
