# Supplementary material for: Semantic design of functional de novo genes from a genomic language model
Source: Nature. 2025 Nov 19;649(8097):749–58. doi: 10.1038/s41586-025-09749-7 (PMC12804078; doi:10.1038/s41586-025-09749-7)
Supplement: Supplementary file 1 — This file contains Supplementary Figures 1 and 2. Supplementary Figure 1: Genomic context of closest sequence matches of EvoAT1-4 and EvoAcr3; Supplementary Figure 2: Uncropped phage plaque images for Acr protection assay. [file 41586_2025_9749_MOESM1_ESM.pdf]

nature portfolio

https://doi.org/10.1038/s41586-025-09749-7

Supplementary information

Semantic design of functional de novo genes from a genomic language model

In the format provided by the
authors and unedited

Semantic design of functional *de novo* genes from a genomic language model

Aditi T. Merchant[1,4], Samuel H. King[1,4], Eric Nguyen[1,4], and Brian L. Hie[2,3,4,*]

[1]Department of Bioengineering, Stanford University, Stanford, CA; [2]Department of Chemical Engineering, Stanford University, Stanford, CA; [3]Stanford Data Science, Stanford University, Stanford, CA; [4]Arc Institute, Palo Alto, CA.
*Correspondence: brianhie@stanford.edu

Supplementary information

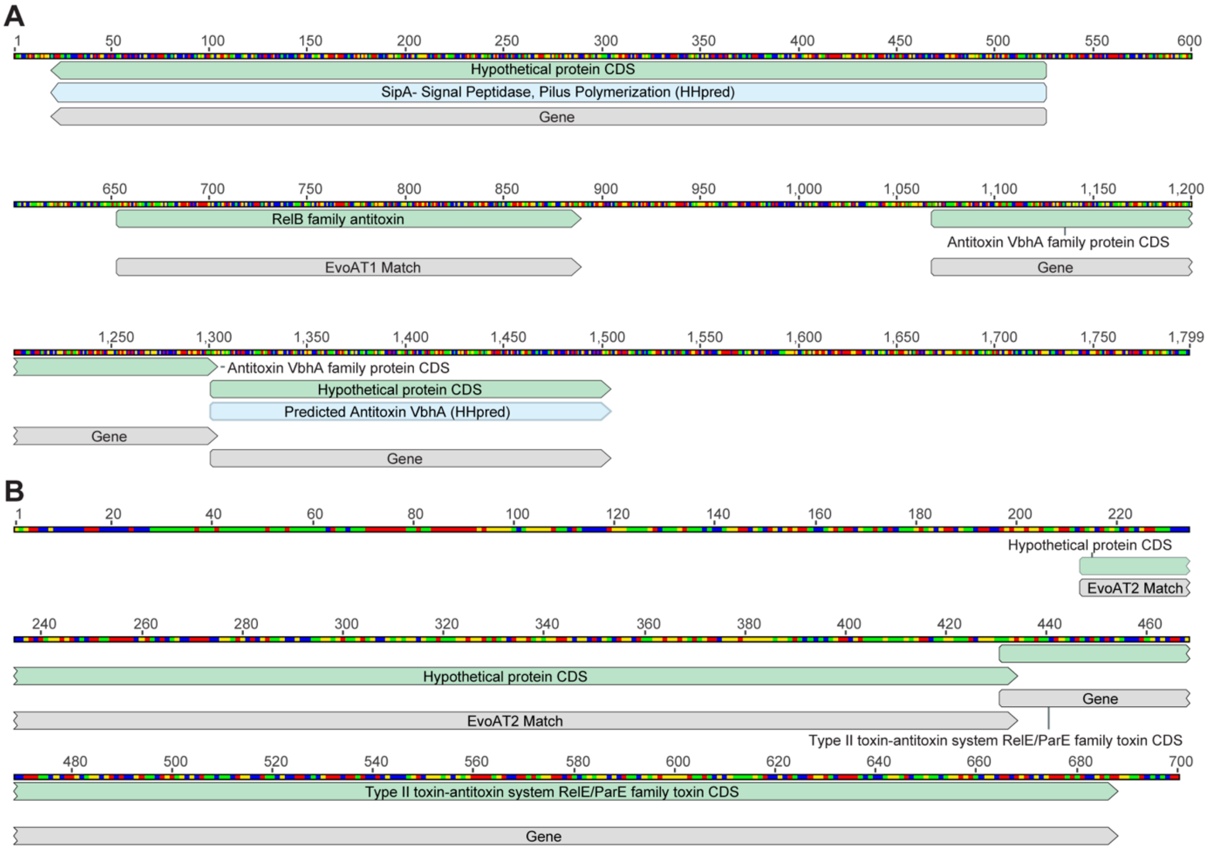
A
Hypothetical protein CDS
SipA- Signal Peptidase, Pilus Polymerization (HHpred)
Gene
RelB family antitoxin
EvoAT1 Match
Antitoxin VbhA family protein CDS
Gene
Antitoxin VbhA family protein CDS
Hypothetical protein CDS
Predicted Antitoxin VbhA (HHpred)
Gene
Gene
B
Hypothetical protein CDS
EvoAT2 Match
Hypothetical protein CDS
Gene
EvoAT2 Match
Type II toxin-antitoxin system RelE/ParE family toxin CDS
Type II toxin-antitoxin system RelE/ParE family toxin CDS
Gene

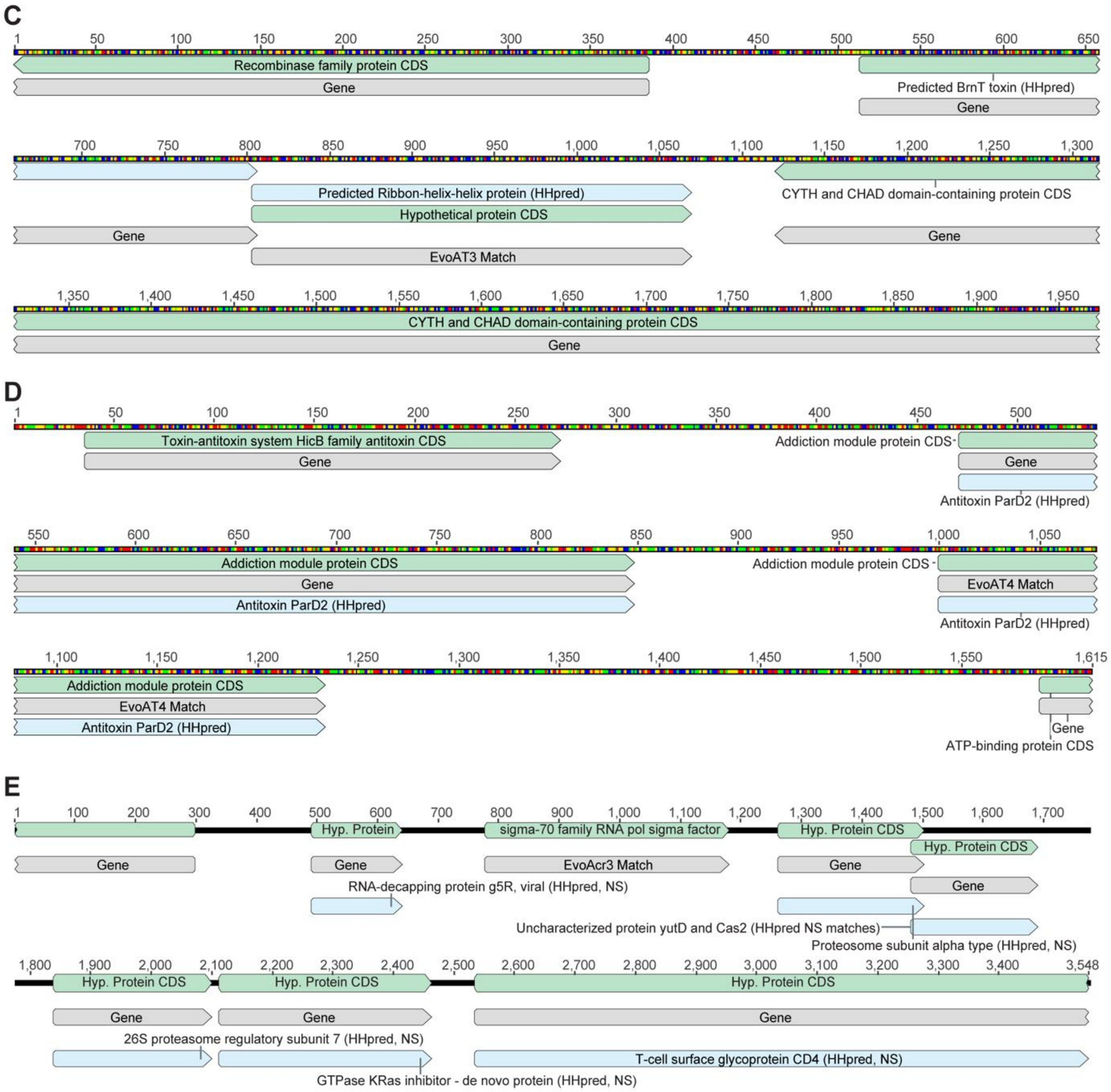

Supplementary Figure 1: Genomic context of closest sequence matches of EvoAT1-4 and EvoAcr3. (A-D) Genes known and predicted to be related to toxin-antitoxin systems can be found in the immediate vicinity of the closest sequence identity matches for EvoAT1-4. **(E)** The closest sequence match for EvoAcr3, a sigma-70 RNA pol sigma factor, is surrounded by primarily uncharacterized proteins that are not known to be anti-CRISPRs. NS, non-significant.

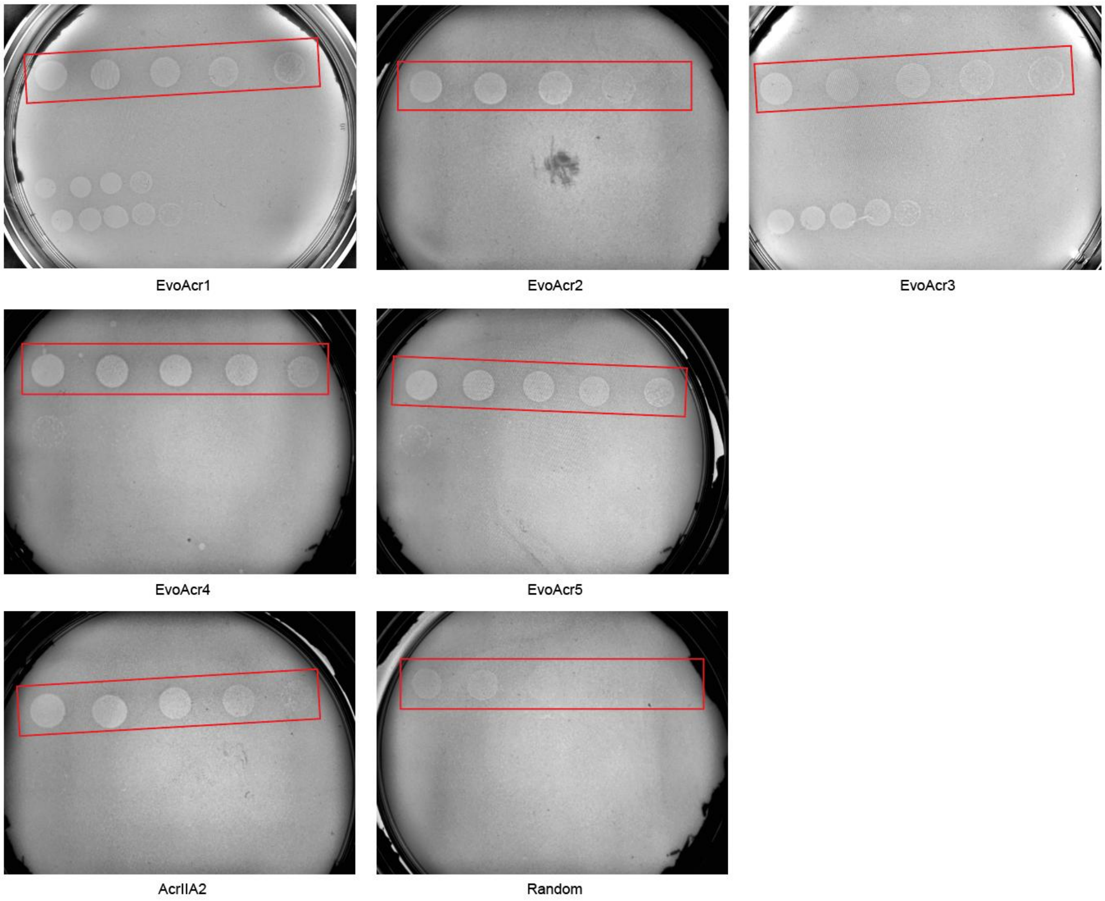

Supplementary Figure 2. Uncropped phage plaque images for Acr protection assay. Uncropped phage plaque images depicting formation of plaques in response to EvoAcr1-5, AcrIIA2, and a random sequence.
