# Supplementary material for: Semantic design of functional de novo genes from a genomic language model
Source: Nature. 2025 Nov 19;649(8097):749–58. doi: 10.1038/s41586-025-09749-7 (PMC12804078; doi:10.1038/s41586-025-09749-7)
Supplement: Supplementary file 2 — Reporting Summary [file 41586_2025_9749_MOESM2_ESM.pdf]

nature portfolio

Corresponding author(s): Brian L. Hie

Last updated by author(s): Sep 21, 2025

Reporting Summary

Nature Portfolio wishes to improve the reproducibility of the work that we publish. This form provides structure for consistency and transparency in reporting. For further information on Nature Portfolio policies, see our Editorial Policies and the Editorial Policy Checklist.

Statistics

For all statistical analyses, confirm that the following items are present in the figure legend, table legend, main text, or Methods section.

n/a	Confirmed	
☐	☒	The exact sample size (*n*) for each experimental group/condition, given as a discrete number and unit of measurement
☐	☒	A statement on whether measurements were taken from distinct samples or whether the same sample was measured repeatedly
☐	☒	The statistical test(s) used AND whether they are one- or two-sided<br>*Only common tests should be described solely by name; describe more complex techniques in the Methods section.*
☐	☒	A description of all covariates tested
☐	☒	A description of any assumptions or corrections, such as tests of normality and adjustment for multiple comparisons
☐	☒	A full description of the statistical parameters including central tendency (e.g. means) or other basic estimates (e.g. regression coefficient) AND variation (e.g. standard deviation) or associated estimates of uncertainty (e.g. confidence intervals)
☐	☒	For null hypothesis testing, the test statistic (e.g. *F*, *t*, *r*) with confidence intervals, effect sizes, degrees of freedom and *P* value noted<br>*Give P values as exact values whenever suitable.*
☐	☒	For Bayesian analysis, information on the choice of priors and Markov chain Monte Carlo settings
☐	☒	For hierarchical and complex designs, identification of the appropriate level for tests and full reporting of outcomes
☐	☒	Estimates of effect sizes (e.g. Cohen's *d*, Pearson's *r*), indicating how they were calculated

Our web collection on statistics for biologists contains articles on many of the points above.

Software and code

Policy information about availability of computer code

Data collection	Prompts were fetched using information from AcrDB, TADB 3.0, UniProt and NCBI GenBank via the UniProt Rest API and NCBI Entrez E-utilities. The original Evo model inference code used for sampling was adapted from Nguyen et al. 2024 (https://github.com/evo-design/evo). All code was written in Python (v3.11.8).
Data analysis	To evaluate generated sequences, we used Prodigal (v2.6.3), MAFFT (v7.526), DustMasker (v2.14.1 +galaxy2), SegMasker (v2.14.l+galaxy2), NCBI BLAST against the non-redundant protein database (https://blast.ncbi.nlm.nih.gov/Blast.cgi, run on July 14, 2025), MMseqs2 (Steinegger and Soding, 2017, v15.6f452), Rfam (Ontiveros-Palacios et al., 2025, v15.0), Pfam-A (Paysan-Lafosse et al., 2024, v35.0) the HHpred webserver (https://toolkit.tuebingen.mpg.de/tools/hhpred, PDB_mmCIF70_25_May), the Dali webserver (ekhidna.biocenter.helsinki.fi/dali_server/, Holm et al., 2022, v22.5.2025), PaCRISPR (https://pacrispr.erc.monash.edu/server.jsp, Wang et al., 2020) and the Foldseek webserver (https://search.foldseek.com/search, van Kempen et al., 2023, v10-941cd33). We also utilized code from ESMFold (https://github.com/facebookresearch/esm, Lin et al., 2023, v1.0.3) and pDockQ calculation code from Bryant et al. 2022 (https://gitlab.com/ElofssonLab/FoldDock, version from Apr 18, 2023). SynGenome analysis was done with the assistance of ScanPy (Wolf et al., 2018, v1.10.3), the HHsuite (Finn et al., 2011, v 3.3.0), Pfam-A (Paysan-Lafosse et al., 2024, v35.0), igraph (Csardi et al., 2006, v0.11.6), and SciPy (v1.11.4). All code was written in Python (v3.11.8).

For manuscripts utilizing custom algorithms or software that are central to the research but not yet described in published literature, software must be made available to editors and reviewers. We strongly encourage code deposition in a community repository (e.g. GitHub). See the Nature Portfolio guidelines for submitting code & software for further information.

Data

Policy information about availability of data

All manuscripts must include a data availability statement. This statement should provide the following information, where applicable:

- Accession codes, unique identifiers, or web links for publicly available datasets
- A description of any restrictions on data availability
- For clinical datasets or third party data, please ensure that the statement adheres to our policy

SynGenome is explorable and searchable at https://evodesign.org/syngenome/. Raw data has been deposited to Hugging Face datasets at https://huggingface.co/datasets/evo-design/syngenome-uniprot. Protein structures predicted by ESMFold for ~3.7 million genes in SynGenome have been deposited to Hugging Face datasets at https://huggingface.co/datasets/evo-design/syngenome-protein-structures. DNA, RNA, and protein sequences used during our validation experiments are available in Supplementary Data 1. Data from our bioinformatic analyses of our generated sequences can be found in Supplementary Data 2-5. Sequence prompts for gene and operon completion evaluations, anti-CRISPR generation, toxin-antitoxin generation, and SynGenome generation were derived from NCBI GenBank (https://www.ncbi.nlm.nih.gov/genbank/) , AcrDB (https://bcb.unl.edu/AcrDB/), TADB 3.0 (https://bioinfo-mml.sjtu.edu.cn/TADB3/index.php), and Uniprot (https://www.uniprot.org/), as described in Methods. OpenGenome is available at https://huggingface.co/datasets/LongSafari/open-genome. All newly created materials are available upon reasonable request to the corresponding author.

Research involving human participants, their data, or biological material

Policy information about studies with human participants or human data. See also policy information about sex, gender (identity/presentation), and sexual orientation and race, ethnicity and racism.

Reporting on sex and gender	Not applicable to this study.
Reporting on race, ethnicity, or other socially relevant groupings	Not applicable to this study.
Population characteristics	Not applicable to this study.
Recruitment	Not applicable to this study.
Ethics oversight	Not applicable to this study.

Note that full information on the approval of the study protocol must also be provided in the manuscript.

Field-specific reporting

Please select the one below that is the best fit for your research. If you are not sure, read the appropriate sections before making your selection.

☒ Life sciences ☐ Behavioural & social sciences ☐ Ecological, evolutionary & environmental sciences

For a reference copy of the document with all sections, see nature.com/documents/nr-reporting-summary-flat.pdf

Life sciences study design

All studies must disclose on these points even when the disclosure is negative.

Sample size	For experimentally tested anti-CRISPRs and toxin-antitoxins, the number of screened candidates is listed in the methods and main text. As described in methods, experimentally tested designs were chosen based on passing all in silico filtering metrics, their level of sequence novelty (with preference for more novel sequences), and available experimental resources and throughput. As we were able to identify functional proteins from the initial batches of experimentally screened generations, the sample sizes chosen were sufficient to demonstrate our approach.
Data exclusions	Generated sequences used for experimental validation were filtered according to in silico sequence (length, complexity, sequence similarity) and/or structure (ESMFold and AlphaFold 3 metric cutoffs) metrics, as documented in the methods section. SynGenome generations were filtered to remove low-complexity, highly repetitive sequences in an effort to reduce the number of low-quality model artifacts in this public resource, as described in the methods.
Replication	All experiments were conducted with n=3 biological replicates (type II toxin-antitoxin and anti-CRISPR) or n=6 biological replicates (type III toxin-antitoxin), with replicates defined as independent bacterial transformations for liquid culture screens and independent transformations followed by independent phage plaque experiments for the phage plaque assays. All attempts at replication were successful.
Randomization	None of our in silico or in vitro experiments required group allocation. To normalize the number of sequences used for comparisons between different prompts, different generation batches, or natural versus generated sequences, samples of PaCRISPR-passing Acrs, sequence completion generations, SynGenome, and OpenGenome were selected using Python's random module, as described in methods.

	Randomization was not applicable to any of our other in vitro or in silico experiments as there were no animals, human subjects, or other groups were involved that would necessitate random allocation or covariate control.
Blinding	All experiments were conducted in a controlled experimental setting using objective data measurement instruments, resulting in no need for blinding.

Reporting for specific materials, systems and methods

We require information from authors about some types of materials, experimental systems and methods used in many studies. Here, indicate whether each material, system or method listed is relevant to your study. If you are not sure if a list item applies to your research, read the appropriate section before selecting a response.

Materials & experimental systems

n/a	Involved in the study
☒	☐ Antibodies
☒	☐ Eukaryotic cell lines
☒	☐ Palaeontology and archaeology
☒	☐ Animals and other organisms
☒	☐ Clinical data
☒	☐ Dual use research of concern
☒	☐ Plants

Methods

n/a	Involved in the study
☒	☐ ChIP-seq
☒	☐ Flow cytometry
☒	☐ MRI-based neuroimaging

Plants

Seed stocks	Not applicable to this study.
Novel plant genotypes	Not applicable to this study.
Authentication	Not applicable to this study.
